# Dissociable mesolimbic dopamine circuits control responding triggered by alcohol-predictive discrete cues and contexts

**DOI:** 10.1038/s41467-020-17543-4

**Published:** 2020-07-28

**Authors:** Milan D. Valyear, Iulia Glovaci, Audrey Zaari, Soraya Lahlou, Ivan Trujillo-Pisanty, C. Andrew Chapman, Nadia Chaudhri

**Affiliations:** Center for Studies in Behavioral Neurobiology, Department of Psychology, Concordia University, Montreal, QC Canada

**Keywords:** Motivation, Neural circuits, Reward

## Abstract

Context can influence reactions to environmental cues and this elemental process has implications for substance use disorder. Using an animal model, we show that an alcohol-associated context elevates entry into a fluid port triggered by a conditioned stimulus (CS) that predicted alcohol (CS-triggered alcohol-seeking). This effect persists across multiple sessions and, after it diminishes in extinction, the alcohol context retains the capacity to augment reinstatement. Systemically administered eticlopride and chemogenetic inhibition of ventral tegmental area (VTA) dopamine neurons reduce CS-triggered alcohol-seeking. Chemogenetically silencing VTA dopamine terminals in the nucleus accumbens (NAc) core reduces CS-triggered alcohol-seeking, irrespective of context, whereas silencing VTA dopamine terminals in the NAc shell selectively reduces the elevation of CS-triggered alcohol-seeking in an alcohol context. This dissociation reveals new roles for divergent mesolimbic dopamine circuits in the control of responding to a discrete cue for alcohol and in the amplification of this behaviour in an alcohol context.

## Abstract (web summary)

Alcohol craving can be enhanced by alcohol-associated cues and by alcohol-associated contexts. Here the authors investigate the role of the ventral tegmental area (VTA)-to-nucleus accumbens (NAc) core and VTA-to-NAc shell circuits in mediating these distinct aspects of alcohol seeking behaviour in rats.

## Introduction

The environmental context in which learning occurs can gate the future expression of that learning^1,2^. Moreover, the influence of context on learning and cognition can be life-changing for people with substance use disorder^3,4^. In support of this idea, preclinical studies show that contexts associated with drug availability can trigger relapse-like renewal of extinguished drug-seeking behaviour^5–7^. These data bolster a critical role for context in substance use disorders^8^, and extend a central hypothesis in the addiction field, which is that environmental stimuli can prompt drug-seeking, -taking and relapse^3,9,10^. This hypothesis originated in studies on the incentive and motivational properties of discrete drug-predictive cues, which in humans are environmental stimuli that occur in close temporal proximity with drug use (e.g., smell or taste of alcohol). In preclinical studies these stimuli are modelled by pairing drug delivery with a brief, non-drug stimulus (e.g., light and/or tone)^11^. Modern theories on substance use disorder acknowledge that alongside discrete drug cues, drug-associated contexts facilitate problematic substance use^12–14^.

In animal models, a context can be created by introducing a stable configuration of multimodal stimuli to the testing chamber^5,13^. Interestingly, studies on learning and memory processes that use such contexts show that context is a crucial determinant of how animals respond to discrete cues^1,15–17^. This conclusion is germane to people with substance use disorders because discrete drug cues are usually embedded within drug contexts, and the simultaneous occurrence of both discrete drug cues and drug contexts produces stronger drug craving and more rapid initiation of drug use than either one alone^18^.

To understand how discrete drug cues and drug contexts intersect to promote drug-seeking behaviour and to study the neural processes that underpin these interactive effects, we developed an animal model to test how rats respond to a discrete Pavlovian conditioned stimulus (CS) that predicted alcohol in different contexts. We found that CS-triggered approach and entry into a fluid port (i.e., CS-triggered alcohol-seeking) was significantly elevated in an alcohol context, compared to either a novel context or an equally familiar context where alcohol had not previously been consumed^19–21^. Here, we sought deeper insight into the psychological processes underlying elevation of CS-triggered alcohol-seeking by an alcohol context. To determine if this effect was transient or persistent, we examined CS-triggered alcohol-seeking in both an alcohol context and a familiar, neutral context across multiple sessions in the absence of alcohol delivery. After the influence of the alcohol context on CS-triggered alcohol-seeking had subsided, we determined if this context could still impact responding to the CS in a relapse test.

Dopamine is critical for the instantiation^22–24^ and expression^25^ of cue-reward learning, and plays an important role in behaviour motivated by alcohol^26^. The dopamine system is also engaged by context, as context can modulate how ventral tegmental area (VTA) dopamine neurons fire in response to discrete cues^27^. Exposure to alcohol-associated contexts evokes dopamine release in the nucleus accumbens^28^ (NAc), a key nucleus in brain reward circuitry^29^ that is densely innervated by dopaminergic neurons originating in the VTA^22,29–31^. Moreover, the NAc core and shell subregions are hypothesized to have dissociable roles in behaviour motivated by discrete cues and contexts, respectively^32–34^.

This dissociation is supported by instrumental conditioning studies that directly compared NAc core and shell function in the same behavioural task. Functional inactivation^35,36^ or excitotoxic lesion^37,38^ of the NAc core, but not shell, reduced operant responding that was reinforced by discrete cues. Behaviour motivated by context was reduced by functional inactivation^39^ or pharmacological interference of glutamatergic^40^ or dopaminergic^41^ neurotransmission within the NAc shell. Interestingly, the same manipulations in the NAc core also reduced behaviour in these context-dependent tasks^39–41^, which is inconsistent with the aforementioned dissociation. However, this result would be predicted if the task explicitly or inadvertently included a salient, discrete cue (e.g. a lever).

Perhaps the best evidence for this dissociation in NAc function comes from a study showing that antagonism of dopamine D1-like receptors in the NAc shell, but not core, reduced context-induced renewal of heroin-seeking^32^. These results corroborate a role for the NAc shell and its afferent and efferent connections in mediating the impact of context on operant drug-seeking^42–45^. In that same study, the D1-like receptor antagonist in the NAc core, but not shell, reduced responding reinforced by a discrete cue^32^. Importantly, the cue-induced reinstatement test was conducted in a context that was distinct from the original self-administration context. This manipulation mitigated the contribution of context to test responding, thereby supporting a role for NAc core dopamine in instrumental responding for a discrete cue.

This compelling body of research is bound by two considerations. First, instrumental responding can be supported by the intrinsically reinforcing properties of non-drug stimuli, such as flashes of light^46,47^. Studies showing a role for the NAc core in responding for discrete cues do not report if the non-drug stimuli were intrinsically reinforcing, or if they had acquired the capacity to support instrumental responding because of prior association with primary reinforcement during the self-administration phase. Second, in these studies the discrete cue was always presented in a response-contingent manner, whereas when context was used to trigger behaviour it was presented in a response-independent manner. Thus, the dissociation in NAc core versus shell function could be related to the active (response-contingent) versus passive (response-independent) nature of discrete cue and context presentation^32^. Our behavioural task, described briefly above, circumvents these considerations. Rats are trained to associate a brief auditory CS with alcohol in a specific context and acquisition and expression of this CS-alcohol association can be tracked. At test, CS-triggered alcohol-seeking is assessed in an alcohol context and in a neutral context, and both the CS and context are passively presented.

We found previously that CS-triggered alcohol-seeking required functional activity in the NAc core but not shell, whereas context-induced renewal of CS-triggered alcohol-seeking was reduced by functionally inactivating either subregion^33^. Furthermore, functional inactivation of the NAc shell diminished contextual control over CS-triggered alcohol-seeking^20^. In the present study, we used pharmacology and chemogenetics to first determine if the dopamine system was involved in responding to a CS that predicted alcohol when presented in a familiar context where alcohol had not previously been consumed. Next, we used a validated, circuit-specific chemogenetic approach to test two predictions emerging from the hypothesis that the NAc core and shell subregions have dissociable roles in responding to discrete drug cues and drug contexts, respectively. First, we predicted that if dopamine neurotransmission in the NAc core is necessary for responding to discrete drug cues, then silencing dopaminergic inputs from the VTA to the NAc core would reduce CS-triggered alcohol-seeking regardless of where the CS was experienced. Second, if dopamine in the NAc shell is necessary for context-mediated drug-seeking then silencing dopaminergic inputs from the VTA to the NAc shell would selectively reduce the elevation of CS-triggered alcohol-seeking in an alcohol context, without affecting CS-triggered alcohol-seeking in a neutral context. Our results are consistent with these predictions, and reveal that divergent mesolimbic dopamine circuits control responding to discrete cues for alcohol in a context-dependent manner.

## Results

### An alcohol context invigorates CS-triggered alcohol-seeking

Conditioned approach and entry into a fluid port triggered by a discrete CS that predicted alcohol (i.e., CS-triggered alcohol-seeking) is significantly elevated in a context associated with prior alcohol intake (alcohol context), relative to a familiar, neutral context where alcohol was never consumed^19–21^. To determine if this effect was transient or persistent, we examined CS-triggered alcohol-seeking in an alcohol context and a neutral context across multiple sessions without alcohol delivery (i.e., extinction). Once the behavioural impact of the alcohol context was extinguished, we determined if the alcohol context retained the ability to influence CS-triggered alcohol-seeking in a priming-induced relapse test.

**Fig. 1:**
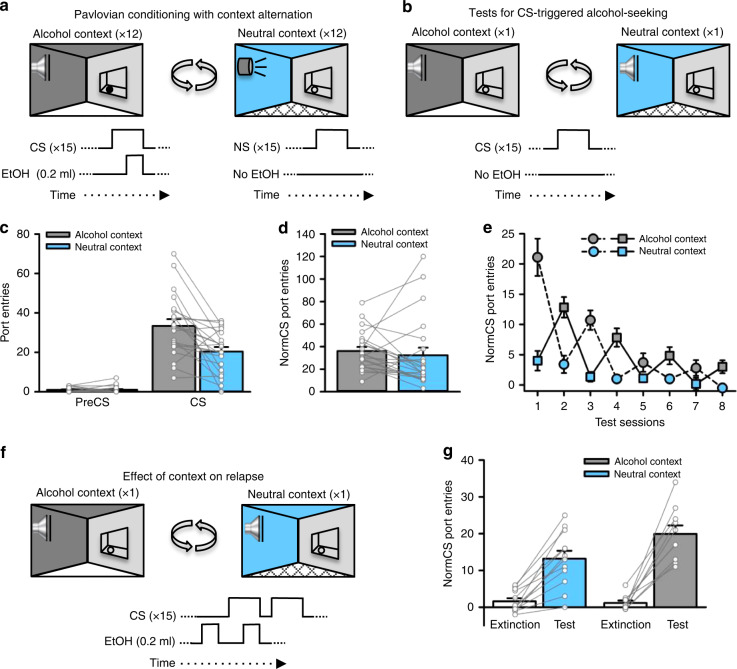
An alcohol context invigorates CS-triggered alcohol-seeking. **a** Rats (*n* = 22) received Pavlovian conditioning sessions every other day in a distinct ‘alcohol context’ where a discrete, conditioned stimulus (CS) was paired with alcohol. On alternating days, rats were exposed to a different, ‘neutral context’ where a neutral stimulus (NS) was presented without alcohol. **b** Port-entries elicited by the CS (i.e., CS-triggered alcohol-seeking) were tested in the alcohol and neutral contexts by presenting the CS without alcohol. **c** At test, CS, but not PreCS, port-entries were elevated in the alcohol context compared to the neutral context [Context × Interval, *F*_(1,20)_ = 14.656, *p* = 0.001, *η*_*p*_^2^ = 0.423; *t*_(21)_ = 3.736, *p* = 0.001, *η*_*p*_^2^ = 0.399]. **d** NonCS port-entries were unaffected by context [Context, *F*_(1,20)_ = 0.363, *p* = 0.554, *η*_*p*_^2^ = 0.018]. **e** We then conducted four additional tests in the alcohol and neutral contexts to determine if the impact of the alcohol context on CS-triggered alcohol-seeking was persistent. Normalized CS port-entries (CS minus PreCS; mean ± s.e.m.) decreased across repeated tests [Session, *F*_(3,30)_ = 18.435, *p* < 0.001, *η*_*p*_^2^ = 0.648], but were elevated in the alcohol context relative to the neutral context [Context, *F*_(1,10)_ = 30.671, *p* < 0.001, *η*_*p*_^2^ = 0.754; Context × Session, *F*_(3,30)_ = 5.885, *p* = 0.003, *η*_*p*_^2^ = 0.370]. Rats received the first test in the alcohol context (dashed line) or neutral context (solid line). **f** We then investigated the impact of context on relapse by presenting a drop of alcohol in the fluid port before and during the first CS presentation to reinstate CS port-entries. Half of the rats received the reinstatement test in the neutral context following their last extinction session in the alcohol context and vice versa. **g** Relative to an averaged baseline of the last two extinction sessions, normalized CS port-entries were reinstated in both contexts [Phase, *F*_(1,20)_ = 108.159, *p* < 0.001, *η*_*p*_^2^ = 0.844], but to a greater extent in the alcohol context than in the neutral context [Phase × Reinstatement Context, *F*_(1,20)_ = 6.037, *p* = 0.023, *η*_*p*_^2^ = 0.232; *t*_(20)_ = 2.112, *p* = 0.047, *η*_*p*_^2^ = 0.182]. Averaged data are mean ± s.e.m. with data from individual rats overlaid on the bar graphs. Data were analysed using RM ANOVA (**c**, **d**, **e**, **g**) and Bonferroni-corrected *t* tests (**c**, **g**). All statistical tests were two-sided. Raw data are available as a supplementary source data file.

Outbred, male Long-Evans rats (*n* = 22) were acclimated to drinking 15% ethanol (alcohol) in their home-cages for 12 sessions (Supplementary Fig. 1a) and then given Pavlovian conditioning sessions in which 15 presentations of a discrete, 10-s auditory CS were paired with alcohol (0.2 ml per CS; 3.0 ml per session) delivered into a fluid port for oral intake. Pavlovian conditioning sessions occurred in a distinct, alcohol context created by visual, olfactory and tactile stimuli in the conditioning chamber, and were alternated daily with sessions in a different, neutral context where alcohol was never available (Fig. 1a). While in the neutral context, half of the rats received presentations of an auditory stimulus that was distinct from their CS (referred to as the neutral stimulus, NS group), whereas the remainder did not (no-NS group). The purpose of the NS was to equate the alcohol and neutral contexts in terms of acoustical salience. Since there were no significant main effects or interactions as a function of this condition, data reported herein were collapsed across group, and an NS was included in the design of all subsequent experiments (see Supplementary Tables 1 and 2 for summaries of all experiments).

Rats learned to selectively enter the fluid port during the CS (Supplementary Figs. 2a, 3a and 4a). Following acquisition, we examined CS port-entries in two counterbalanced tests, one in the alcohol context and one in the neutral context. At test, the CS was presented as in prior Pavlovian conditioning sessions, but without alcohol (Fig. 1b).

CS port-entries at test were higher than port-entries made during the PreCS interval, and were significantly elevated in the alcohol context relative to the neutral context (*p* = 0.001; Fig. 1c, Supplementary Fig. 5a, d, Supplementary Video 1). There was no impact of context on port-entries made between CS trials (NonCS, Fig. 1d), the latency to initiate a CS port entry (Supplementary Fig. 5b), or the total duration of CS port-entries (Supplementary Fig. 5c). Thus, an alcohol context selectively elevated the number of CS port-entries, without affecting other characteristics of the response.

Next, we presented the CS without alcohol in the alcohol and neutral contexts in alternating sessions. Normalized CS port-entries (i.e., CS minus PreCS) decreased across test sessions, but were elevated and maintained at higher levels for longer in the alcohol context (Fig. 1e). We then examined the impact of context on relapse triggered by re-exposure to alcohol. Rats that began extinction in the alcohol context had a relapse test in the neutral context, and vice versa. At test, 0.2 ml of alcohol was delivered into the fluid port 90 s before the first inter-trial interval and then again during the first of 15 CS trials (Fig. 1f). Normalized CS port-entries increased significantly at test, compared to responding averaged across the last two extinction sessions (one in either context), and was higher in the alcohol context than the neutral context (*p* = 0.047; Fig. 1g). Thus, the alcohol context amplified reinstatement in a relapse test conducted after responding in both the alcohol and neutral contexts had diminished to similarly low levels.

Altogether, while CS-triggered alcohol-seeking underwent classical extinction in both the alcohol and neutral contexts, CS port-entries were maintained at a higher level for longer in the alcohol context and were reinstated to a higher level in the alcohol context. These results show the behavioural impact of an alcohol context is persistent.

### VTA dopamine neurons support CS-triggered alcohol-seeking

**Fig. 2:**
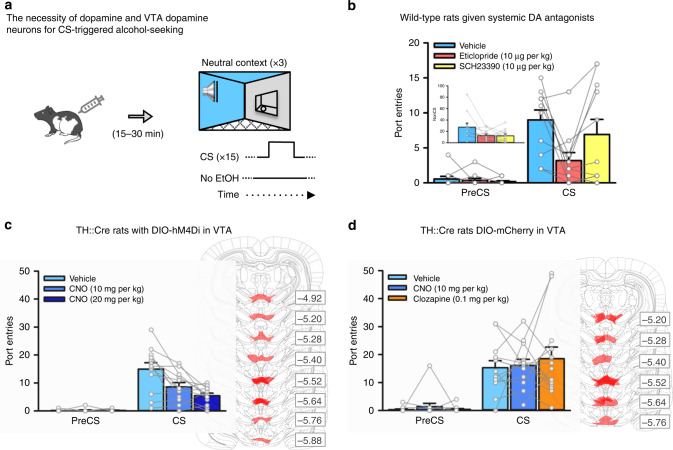
VTA dopamine neurons are necessary for CS-triggered alcohol-seeking. **a** We examined the impact of inhibiting dopamine receptors, or inhibiting VTA dopamine neurons, on CS-triggered alcohol-seeking at test in the neutral context. **b** In a subset (*n* = 11) of wild-type rats from Fig. 1, systemic administration of the dopamine D2-like receptor antagonist eticlopride [Interval × Dopamine Antagonist, *F*_(2, 20)_ = 4.294, *p* = 0.028, *η*_*p*_^*2*^ = 0.300; Bonferroni-corrected *t*_(10)_ = 3.56, *p* = 0.018, *η*_*p*_^2^ = .544], but not the dopamine D1-like receptor antagonist SCH23390 (*p* > 1 after correction), significantly reduced CS port-entries relative to vehicle. NonCS port-entries were also significantly reduced following dopamine receptor antagonist administration relative to vehicle [inset; Dopamine Antagonist, *F*_(2, 20)_ = 4.174, *p* = 0.031, *η*_*p*_^2^ = 0.294]. **c** Next, we used a chemogenetic approach in TH::Cre rats (*n* = 12) that expressed the inhibitory designer receptor, hM4Di, in ventral tegmental area (VTA) dopamine neurons. At test, CS port-entries were significantly reduced compared to vehicle [Interval × CNO Dose, *F*_(2, 22)_ = 10.842, *p* = 0.001, *η*_*p*_^2^ = 0.596] following systemic administration of 10 mg per kg and 20 mg per kg of clozapine-*n*-oxide (CNO) [Bonferroni-corrected *t*_(11)_ = 3.171, *p* = 0.03, *η*_*p*_^2^ = 0.478 for 10 mg per kg, and *t*_(11)_ = 3.644, *p* = 0.012, *η*_*p*_^2^ = 0.547 for 20 mg per kg, relative to vehicle]. Maximal hSyn-DIO-hM4Di-mCherry expression in the VTA for each rat is shown in schematics from the atlas of Paxinos and Watson (2007)^85^. Numbers represent the anterior−posterior coordinate relative to bregma. **d** The impact of the lowest effective dose of CNO, and its parent compound clozapine (0.1 mg per kg), on CS-triggered alcohol-seeking was assessed in TH::Cre rats (*n* = 13) expressing the control DIO-mCherry construct in the VTA. Relative to vehicle, neither 10 mg per kg CNO nor 0.1 mg per kg clozapine significantly affected CS port-entries [Interval × Treatment, *F*_(2, 24)_ = 0.431, *p* = 0.655, *η*_*p*_^2^ = 0.035]. Maximal hSyn-DIO-mCherry expression for each rat is shown to the right. Averaged data are mean ± s.e.m. with data from individual rats overlaid on the bar graphs. Data were analysed using RM ANOVA (**b**–**d**) and Bonferroni-corrected *t* tests (**b**, **c**). All statistical tests were two-sided. Raw data are available as a supplementary source data file.

Functional imaging studies implicate dopamine in cue-induced alcohol craving^48^, suggesting that dopamine is involved in responding to discrete alcohol cues experienced outside drug contexts (i.e., laboratory setting). We tested this hypothesis in our animal model. A subset of rats (*n* = 11) from experiment 1 were retrained in the procedure described above and then CS port-entries without alcohol were tested in a neutral context following subcutaneous injection of saline or a dopamine D1-like (SCH23390, 10 μg per kg) or dopamine D2-like (eticlopride, 10 μg per kg) receptor antagonist (Fig. 2a). Pretreatment with either antagonist reduced NonCS port-entries, relative to vehicle (Fig. 2b, inset). CS port-entries were also reduced by eticlopride (*p* = 0.018), but not by SCH23390 (*p* > 1 after correction), relative to vehicle (Fig. 2b). Thus, dopamine neurotransmission at D1-like and D2-like receptors was required for port-directed behaviour, while CS-triggered alcohol-seeking required D2-like, but not D1-like receptors. A reduction in NonCS port-entries may have been due to locomotor deficits caused by blocking dopamine receptors; however, the same dose of eticlopride used here did not impact locomotor behaviour in an open field^49,50^. Further, port-entries elicited by a CS that was paired with alcohol were unaffected by 10 μg per kg eticlopride^51^.

We then used chemogenetics in TH::Cre rats to evaluate the impact of selectively reducing activity in a subset of VTA dopamine neurons on CS-triggered alcohol-seeking in a neutral context. Naïve TH::Cre male rats (*n* = 12) were microinfused bilaterally into the VTA with the double-floxed inhibitory designer receptor construct^52^ AAV8-hSyn-DIO-hM4Di-mCherry, causing selective expression of the inhibitory designer receptor (hM4Di) in VTA dopamine neurons. This receptor inhibits neuronal firing when bound by the exogenous ligand clozapine-*n*-oxide (CNO)^52,53^. After 12 sessions of home-cage alcohol exposure (Supplementary Fig. 1b) followed by Pavlovian conditioning with context alternation (Supplementary Figs. 2b, 3b and 4b), we tested CS-triggered alcohol-seeking. At test we presented the CS without alcohol in the neutral context 30 min after intraperitoneal injection of vehicle or CNO (10 mg per kg or 20 mg per kg; Fig. 2a)^30,53^. PreCS port-entries were minimal at test, and CS port-entries were reduced in a dose-related manner by 10 mg per kg (*p* = 0.03) and 20 mg per kg (*p* = 0.012) CNO (Fig. 2c). The reduction in CS port-entries was not caused by general decreases in locomotion, as NonCS port-entries were similar across tests (Supplementary Fig. 6a).

**Fig. 3:**
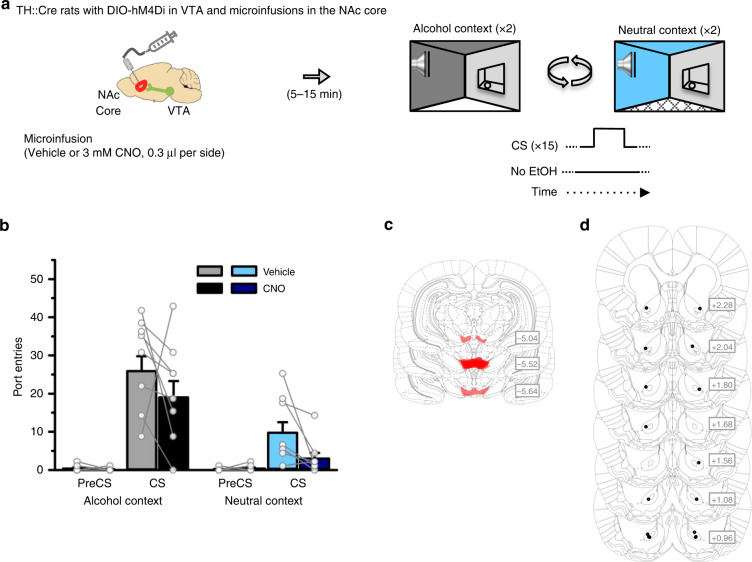
A VTA-to-NAc core dopamine circuit is necessary for CS-triggered alcohol-seeking. **a** TH::Cre rats expressing the inhibitory designer receptor hM4Di in VTA dopamine neurons received Pavlovian conditioning with context alternation, and then CS-triggered alcohol-seeking was tested four times per rat, twice in the alcohol context and twice in the neutral context. Rats received a microinfusion of vehicle or CNO (3 mM, 0.3 μl) into the NAc core (*n* = 8) prior to each test. **b** CS port-entries were elevated at test in the alcohol context relative to the neutral context [Context × Interval, *F*_(1,7)_ = 44.840, *p* < 0.001, *η*_*p*_^2^ = 0.865]. Relative to vehicle, CS port-entries were significantly reduced following CNO microinfusion in the NAc core in both contexts [Treatment × Interval, *F*_(1,7)_ = 5.763, *p* = 0.047, *η*_*p*_^2^ = 0.452; Context × Interval × Treatment, *F*_(1,7)_ = 0.002, *p* = 0.962, *η*_*p*_^2^ < .001]. Clozapine-*n*-oxide microinfusion significantly reduced port-entries during the CS interval (*p* = 0.043) but not during the PreCS interval (*p* > 1 after correction) as indicated by simple main effects analysis of Treatment collapsed across context. Histological results show mCherry expression in the **c** VTA and corresponding injector tip placements in the **d** NAc core. Averaged data are mean ± s.e.m. with data from individual rats overlaid on the bar graphs. Data were analysed using RM ANOVA (**b**) and Bonferroni-corrected simple main effects (**b**). All statistical tests were two-sided. Raw data are available as a supplementary source data file.

**Fig. 4:**
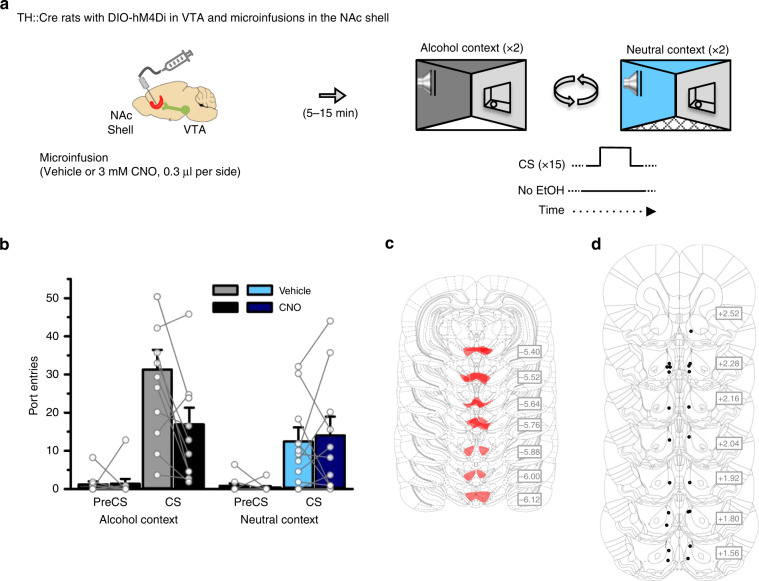
A VTA-to-NAc shell dopamine circuit is necessary for CS-triggered alcohol-seeking in an alcohol context. **a** TH::Cre rats expressing the inhibitory designer receptor hM4Di in VTA dopamine neurons received Pavlovian conditioning with context alternation, and then CS-triggered alcohol-seeking was tested four times per rat, twice in the alcohol context and twice in the neutral context. Rats received a microinfusion of vehicle or CNO (3 mM, 0.3 μl) into the NAc shell (*n* = 11) prior to each test. **b** CS port-entries were elevated at test in the alcohol context relative to the neutral context [Context × Interval, *F*_(1,10)_ = 9.562, *p* = 0.011, *η*_*p*_^2^ = 0.489]. Relative to vehicle, there was no effect of CNO on CS port-entries at test in the neutral context. However, CNO significantly reduced CS port-entries at test in the alcohol context [Context × Interval × Treatment, *F*_(1,10)_ = 5.121, *p* = 0.047, *η*_*p*_^2^ = 0.339; CS port-entries in neutral context, *t*_(10)_ = −0.367, *p* = 0.721, *η*_*p*_^2^ = 0.013; CS Port-entries in alcohol context, *t*_(10)_ = 3.121, *p* = 0.011, *η*_*p*_^2^ = 0.493]. Histological results show mCherry expression in the **c** VTA and corresponding injector tip placements in the **d** NAc shell. Averaged data are mean ± s.e.m. with data from individual rats overlaid on the bar graphs. Data were analysed using RM ANOVA (**b**) and Bonferroni-corrected *t* tests (**b**). All statistical tests were two-sided. Raw data are available as a supplementary source data file.

To control for potential off-target effects of CNO or its reverse-metabolite, clozapine, on CS port-entries in a neutral context, a naïve group of 13 TH::Cre rats received the same surgical, conditioning (Supplementary Fig. 1c, Figs. 2c, 3c and 4c), and test procedures except that they were microinfused with a control virus construct (AAV8-hSyn-DIO-mCherry). Before test, rats received vehicle, the lowest effective dose of CNO (10 mg per kg) from our previous experiment, or clozapine at a dose (0.1 mg per kg) that may result from reverse metabolism of 10 mg per kg CNO^54^, using a within-subject, repeated-measures design. CS port-entries at test in the neutral context were similar following vehicle, CNO, or clozapine (Fig. 2d), as were NonCS port-entries (Supplementary Fig. 6b). In a separate study, CNO (10 mg per kg) had no impact on responding to a CS that predicted sucrose in a neutral context (Supplementary Fig. 7). Thus, suppression of CS-triggered alcohol-seeking produced by chemogenetically inhibiting VTA dopamine neurons could not be accounted for by non-specific effects of systemically injected CNO or its reverse metabolism to clozapine and did not generalize to a sucrose reinforcer. These results support the hypothesis that VTA dopamine neuron activity is necessary for alcohol-seeking triggered by a discrete cue in a neutral context.

### Dopamine circuits support CS-triggered alcohol-seeking

We used circuit-specific chemogenetics to test the predictions that dopaminergic VTA-to-NAc core projections underpin CS-triggered alcohol-seeking regardless of where that CS is experienced, whereas dopaminergic VTA-to-NAc shell inputs are necessary only for the elevation in CS-triggered alcohol-seeking in an alcohol context.

To target VTA dopaminergic projections, we microinfused the AAV8-hSyn-DIO-hM4Di-mCherry construct into the VTA of TH::Cre rats and implanted bilateral cannulae above the NAc core or shell. This approach allowed us to selectively silence dopaminergic VTA-to-NAc core or VTA-to-NAc shell projections by microinfusing CNO into either NAc subregion^30,53,55^. Following home-cage alcohol exposure (Supplementary Fig. 1d, e) and Pavlovian conditioning with context alternation (Supplementary Figs. 2d, e, 3d, e and 4d, e), we tested CS-triggered alcohol-seeking in both the alcohol and neutral context after CNO or vehicle microinfusions. The CNO dose that we used (3 mM; 0.3 μl per hemisphere) did not influence CS port-entries in either the alcohol or neutral contexts in control rats without designer receptors (Supplementary Fig. 8), and had no non-specific effects on motivated behaviour^56,57^.

In eight TH::Cre rats with cannulae targeting the NAc core (Fig. 3a), CS port-entries were elevated over PreCS port-entries and higher at test in the alcohol context, compared to the neutral context (Fig. 3b). Clozapine-*n*-oxide in the NAc core reduced CS port-entries in both the alcohol and neutral contexts (*p* = 0.043; Fig. 3b). NonCS port-entries were unaffected by CNO and showed a trend towards elevation in the alcohol context compared to the neutral context (Supplementary Fig. 6c). A VTA-to-NAc core dopamine circuit was thus necessary for CS-triggered alcohol-seeking, irrespective of the context in which the discrete cue was experienced.

In a separate experiment using 11 TH::Cre rats with cannulae targeting the NAc shell (Fig. 4a), CNO had no effect on CS port-entries at test in the neutral context (*p* = 0.721; Fig. 4b). However, the same manipulation reduced CS port-entries relative to vehicle (*p* = 0.011; Fig. 4b) in the alcohol context. NonCS port-entries were unaffected by CNO treatment, and were significantly higher in the alcohol context than in the neutral context (Supplementary Fig. 6d). These three findings suggest a VTA-to-NAc shell dopamine circuit was not required for alcohol-seeking triggered by a discrete cue per se, but supported the elevation of CS-triggered alcohol-seeking in an alcohol context.

NonCS port-entries in this procedure may reflect a direct, excitatory association between context and alcohol, or unconditioned locomotor or exploratory behaviour. The lack of effect of VTA-to-NAc core or VTA-to-NAc shell inhibition on NonCS port-entries suggests that these projections were not necessary for these behavioural effects.

Together, the results from these two experiments reveal that a VTA-to-NAc core dopamine circuit subserves alcohol-seeking triggered by a discrete CS, while a diverging VTA-to-NAc shell dopamine circuit supports the elevation of CS-triggered alcohol-seeking in an alcohol context.

### Validation of circuit-specific chemogenetic approach

**Fig. 5:**
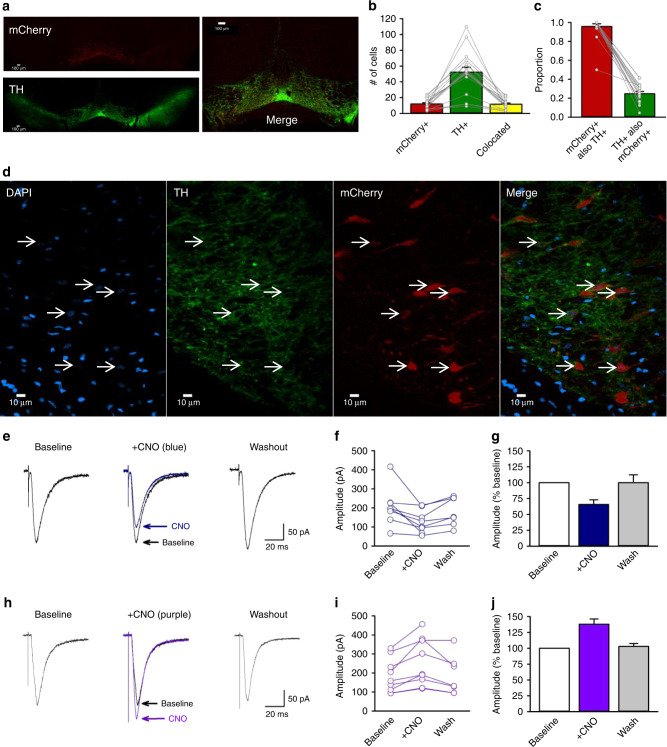
Validation of circuit-specific chemogenetic approach. **a** Representative coronal ×4 optical plane through the VTA showing fluorescence indicative of mCherry (red; top; hM4Di reporter) and TH (green; bottom; Alexa 488), and corresponding merged image (right). **b** The number of cells (*n* = 948 cells across four rats) counted in each 300 × 300 μm optical section. **c** Cell counts indicate the specificity of mCherry expression in TH+ neurons and the proportion of TH+ neurons that were transfected (*n* = 948 cells across four rats). **d** Representative area of a ×40 image used for the analyses in (**b**) and (**c**). Cells with colocalized TH and mCherry signals all had a nucleus indicated by DAPI (blue). **e** Example traces show averaged excitatory post synaptic currents (EPSCs) for an NAc core medium spiny neuron (MSN) before (baseline), during (+CNO), and after (washout) 5 min application of 1 μM CNO to striatal slices containing hm4Di-expressing terminals from VTA dopamine neurons. **f** Peak amplitudes of EPSCs recorded from individual MSNs innervated by hM4Di-expressing dopaminergic terminals (*n* = 8 cells across five rats). **g** Normalized mean EPSC amplitudes for the same group of cells. Clozapine-*n*-oxide reduced EPSC amplitude to 65.7 ± 7.3% of baseline [*n* = 8 cells across five rats; *F*_(2,13)_ = 6.77, *p* = 0.01; Newman−Keuls *p* = 0.01]. Mean EPSC amplitude returned towards baseline values within 20 min of washout (93.9 ± 8.9% of baseline). **h** Example traces show averaged EPSCs for an MSN before (baseline), during (+CNO), and after (washout) 10 min application of CNO to striatal slices containing excitatory hM3Dq-expressing terminals from VTA dopamine neurons. **i** Peak amplitudes of EPSCs recorded from individual MSNs innervated by hm3Dq-expressing terminals (*n* = 9 cells across five rats). **j** Normalized mean EPSC amplitudes for the same group of cells. Clozapine-*n*-oxide significantly increased EPSCs to 137.9 ± 18.8% of baseline levels, [*n* = 9 cells across five rats, *F*_(2,14)_ = 17.84, *p* < 0.001*;* Newman−Keuls *p* < 0.001]. Responses returned to baseline values within 20 min of washout (102.9 ± 4.7% of baseline). Averaged data are mean ± s.e.m. Data were analysed using RM ANOVA (**g**, **j**) and Newman−Keuls post hoc tests (**g**, **j**). All statistical tests were two-sided. Raw data are available as a supplementary source data file.

We validated the selectivity of Cre for TH-positive neurons, and the transfection efficiency of the AAV8-hSyn-DIO-hM4Di-mCherry construct in TH::Cre rats^52,58^. Brains from four TH::Cre^+/−^ rats that received home-cage alcohol exposure and Pavlovian conditioning with context alternation were immunohistochemically labelled for TH and an amplified mCherry signal. In a single optical plane through the VTA at approximately bregma −5.5 mm (Fig. 5a, d), 12.0 ± 1.5 cells were mCherry positive, 52.3 ± 6.3 were TH positive, and 11.6 ± 1.5 were colocalized (Fig. 5b). These counts produced an average selectivity of mCherry expression for TH-positive cells of 95.8% and a transfection efficiency of 24.8% (Fig. 5c), which are comparable to published studies^22,30,53,58^. We also observed mCherry-expressing TH-positive processes in the NAc (Supplementary Figs. 9, 10, and 11).

We conducted in vitro intracellular recordings of excitatory synaptic potentials in NAc core medium spiny neurons (MSNs) to validate the ability of CNO to inhibit hM4Di-expressing terminals of VTA dopamine neurons in the region where our in vivo manipulations occurred. The NAc core showed strong mCherry fluorescence in the region of recorded neurons (Supplementary Fig. 11b, e). Cells were identified as MSNs based on characteristic features of depolarization^59,60^ and action potential waveforms (Supplementary Fig. 11c).

CNO reduced the amplitude of excitatory postsynaptic currents (EPSCs) to 65.7 ± 7.3% of baseline levels (*p* = 0.01; Fig. 5e–g). Thus, activation of inhibitory hM4Di designer receptors in terminals of VTA neurons in the NAc core inhibited excitatory postsynaptic MSN responses.

To confirm that the reduction in MSN EPSCs was the result of CNO actions on designer receptors, we conducted a parallel analysis in NAc core slices that contained VTA dopamine terminals expressing the excitatory designer receptor, hM3Dq. We predicted that CNO, acting on hM3Dq-expressing dopamine terminals in the NAc core, would increase the amplitude of MSN EPSCs, as dopamine has been reported to enhance MSN EPSCs in the NAc^61,62^. Accordingly, CNO significantly increased EPSCs to approximately 137.9 ± 18.8% of baseline levels, (*p* < 0.001*;* Fig. 5h–j). Thus, activation of excitatory hM3Dq designer receptors upon terminals of VTA neurons in the NAc core increased excitatory postsynaptic MSN responses.

## Discussion

We show that an alcohol context elevated CS-triggered alcohol-seeking, and this effect persisted across repeated sessions, and re-emerged after the motivational properties of the alcohol context had been extinguished. Ventral tegmental area dopamine neurons were required for CS-triggered alcohol-seeking in a neutral context, and VTA dopamine input to the NAc core was necessary for CS-triggered alcohol-seeking regardless of where the CS was experienced. Conversely, VTA dopamine input to the NAc shell was not necessary for responding to the discrete CS, but was necessary for the elevation of CS-triggered alcohol-seeking in an alcohol context. This neural dissociation refines our understanding of how the mesolimbic dopamine system regulates behavioural responses to discrete, alcohol-predictive cues in different contexts.

The number of CS port-entries was higher at test in an alcohol context compared to a neutral context; however, there was no impact of context on latency to respond to the CS or the total duration of CS port-entries. These results suggest that a drug context can influence the vigour of conditioned responding to discrete drug cues, but may not impact other features of motivation captured by changes in latency or duration. Interestingly, in a recent study, cigarette smokers exhibited higher craving after exposure to pictures of discrete smoking cues (e.g., a lit cigarette in an ashtray) that were juxtaposed against projected images of smoking contexts, compared to either cue type alone^18^. Smokers were also faster to light up a cigarette after concurrent exposure to discrete and contextual smoking cues, showing that the combination of these types of environmental signals for cigarette smoking accelerated the onset of smoking behaviour.

We found that an alcohol context persistently influenced CS-triggered alcohol-seeking. In the absence of alcohol, CS port-entries extinguished in both contexts; however, there was resistance to extinction in the alcohol context, where CS port-entries remained elevated longer than in the neutral context. Moreover, alternating tests across context produced a saw-tooth pattern of responding, such that CS port-entries increased and then dropped in the alcohol and neutral contexts, respectively. This pattern of responding showcases the remarkable ability of context to control CS-triggered alcohol-seeking. Finally, reinstatement produced by an alcohol prime was significantly higher in the alcohol context than in the neutral context. This effect was unexpected, as rats had stopped discriminating between contexts prior to test. Since the alcohol prime was a different stimulus from the auditory CS, this result suggests that extinguishing one cue across contexts does not prevent a different, non-extinguished cue from prompting a return of alcohol-seeking behaviour, with this effect amplified in an alcohol context.

When considering translational links between the present data and alcohol use disorder, it should be noted that rats in our study drank low levels of alcohol during the home-cage alcohol exposure phase and Pavlovian conditioning. However, home-cage alcohol intake at levels comparable to those in the present study can alter the motivational underpinnings of instrumental behaviour, rendering goal-directed responding habitual^63^. Also, ingested doses of alcohol within the range of what we report during Pavlovian conditioning result in detectable blood levels of alcohol^64^, suggesting that they produce pharmacological effects. Testing the effect of context on CS-triggered alcohol-seeking in alcohol-dependent rats as well as in female rats are two immediate future directions. Nonetheless, the present finding that CS-triggered alcohol-seeking was persistently elevated in an alcohol context, which we report in male non-dependent rats, is likely relevant for people who drink but have not yet developed problematic drinking behaviour. This view is based on research in college students without an alcohol use disorder, who showed more alcohol-related cognitions and elevated alcohol intake in a bar context compared to a neutral, laboratory environment^65^.

The rat VTA contains a large population of dopamine cell bodies that project to diverse striatal and prefrontal targets^22,31,66^. Ventral tegmental area dopamine cell bodies are organized along a medio-lateral gradient, with relatively medial cells projecting predominantly to medial areas of the striatum, including the NAc shell, and more lateral areas projecting to the NAc core^22,29^. We observed designer receptor expression encompassing the anterior−posterior and medio-lateral expanse of VTA dopamine neurons projecting to both the NAc core and shell, with relatively minimal expression in the substantia nigra. While our transfection was highly selective (95.8%), only a relatively small proportion of TH-positive neurons (24.8%) were transfected.

After visualizing mCherry-expressing axons in the NAc, we conducted in vitro electrophysiological experiments to verify that CNO acting on dopamine terminals expressing designer receptors could control NAc neuronal activity. Clozapine-*n*-oxide either decreased or increased the amplitude of EPSCs onto MSNs dependent upon inhibitory or excitatory designer receptors, respectively. This bidirectional effect corroborates evidence that CNO acting on designer receptors on dopaminergic inputs to the NAc can modulate evoked dopamine^30^. It is consistent with at least three mechanisms by which dopamine can influence striatal MSN excitability. First, dopamine can act synergistically at D1 and D2 receptors on MSNs to enhance MSN activity^67^. Second, activation of designer receptors may directly modulate evoked EPSCs by increasing or decreasing co-release of glutamate from VTA dopamine terminals^68,69^. Third, dopamine release from VTA terminals in the NAc can act on corticostriatal terminals to enhance glutamatergic input onto NAc MSNs^70,71^.

Although VTA dopamine neurons are implicated in learning via prediction error^23,24^, no study has directly examined their contribution to CS-triggered alcohol-seeking. We found that blocking dopamine D2-like receptors reduced CS and NonCS port-entries, whereas blocking dopamine D1-like receptors reduced only NonCS port-entries. While systemically administered antagonists can act on multiple, potentially competing dopaminergic circuits, these results implicate the dopamine system in responding to a discrete alcohol CS experienced in a neutral context.

Stronger support for a role of the dopamine system in alcohol-seeking driven by discrete cues was provided by the finding that chemogenetically silencing VTA dopamine neurons reduced CS-triggered alcohol-seeking in a neutral context. This result corroborates data suggesting a role for cholinergic^72^ and glutamatergic^73^ signalling within the VTA in cue conditioning with alcohol. Importantly, neither the lowest effective CNO dose in this experiment nor a dose of clozapine that could have been produced through reverse metabolism of CNO impacted CS-triggered alcohol-seeking in rats that did not express designer receptors. Additionally, the lowest CNO dose that effectively reduced CS-triggered alcohol-seeking had no effect on CS-triggered sucrose-seeking. This result may be explained by high redundancy in the neural circuitries that support responding for natural food reinforcers^74^. Our silencing of a modest proportion of VTA dopamine neurons (~25%; Fig. 5c) may have been sufficient to disrupt CS-triggered alcohol- but not sucrose-seeking.

Chemogenetically silencing VTA inputs to the NAc core reduced CS-triggered alcohol-seeking, regardless of where the CS was presented, suggesting that this dopaminergic input to the NAc core is critical for responding to discrete cues that predict alcohol. This result is consistent with the finding that non-contingent presentation of a cocaine-predictive discrete cue increased dopamine release in the NAc core, but not shell^75^. The exact nature of the involvement of the VTA-to-NAc core circuit in CS-triggered alcohol-seeking might arise from the NAc core being necessary for orchestrating behaviour in response to the best predictors of reinforcement. Supporting this idea, the development of dopamine transients in the NAc core are time locked to the onset and offset of food-predictive cues^76^, and track closely with the earliest reliable predictor of reinforcement^77^. Remarkably, pairing a cue with optogenetic stimulation of VTA dopamine neurons or their projections to the NAc core, but not the NAc shell, leads to the cue acquiring incentive motivational properties^22^. This finding underscores a central role for a VTA-to-NAc core dopamine circuit in Pavlovian learning about discrete cues^22^, and supports earlier observations of a role for dopaminergic innervation of the NAc core in Pavlovian conditioned approach behaviour^25,76,78^.

Silencing VTA dopamine inputs to the NAc shell had no effect on CS-triggered alcohol-seeking in a neutral context, but selectively reduced the elevation of this behaviour in an alcohol context. This behavioural dissociation supports the prediction that a VTA-to-NAc shell dopamine circuit is not necessary for responding to the CS per se, but rather mediates the capacity of the alcohol context to elevate CS-triggered alcohol-seeking. This finding supports an emerging hypothesis that the NAc shell is an important hub in neural circuits that mediate the impact of context on drug-seeking behaviour^33,35,44,45,79^. Interestingly, optogenetic inhibition of NAc shell MSNs projecting onto VTA GABAergic interneurons results in a net inhibition of VTA dopamine neurons, and this reduction in dopamine release is associated with attenuation of context-induced renewal of alcohol-seeking in an operant conditioning procedure^79^. We found that chemogenetically silencing dopamine terminals in the NAc core reduced the strength of synaptic inputs onto MSNs. A similar reduction in dopaminergic inputs to the NAc shell may have reduced the excitability of MSNs that project back onto GABAergic interneurons in the VTA^79,80^, contributing to inhibition within the VTA that may regulate the impact of drug contexts on drug-seeking behaviour.

The behavioural dissociation we report between dopaminergic VTA-to-NAc core and VTA-to-NAc shell projections is unlikely to have been caused by CNO diffusing from one ventral striatal subregion to the next, as we did not detect CNO in microdialysis samples obtained from the NAc shell after a 0.3-μl microinfusion of CNO in the NAc core, despite achieving a quantitation limit of 0.3 nM (Supplementary Fig. 12). Additionally, CNO microinfused into the NAc did not impact CS port-entries in rats without designer receptors (Supplementary Fig. 8).

The VTA-to-NAc core and VTA-to-NAc shell experiments were conducted consecutively in separate groups of rats. There was a significant effect of context on NonCS port-entries in the VTA-to-NAc shell but not VTA-to-NAc core experiment and modestly higher levels of CS port-entries at test following vehicle in the VTA-to-NAc shell experiment. To evaluate these potential cohort differences, we calculated a difference score for CS port-entries in the alcohol and neutral contexts under vehicle conditions for each experiment. This score accounts for differences in baseline levels of responding across experiments by measuring changes in CS port-entries produced by context irrespective of the magnitude of overall responding. According to this score, CS port-entries were elevated similarly in the alcohol context in the VTA-to-NAc shell (18.82 ± 4.89) and VTA-to-NAc core (16.13 ± 4.27) experiments.

In conclusion, we show that context can have a robust and persistent influence on responding to a discrete, alcohol-predictive cue. Distinct mesolimbic dopamine circuits from the VTA-to-NAc core and VTA-to-NAc shell control alcohol-seeking triggered by discrete cues and the amplification of this behaviour by context, respectively. This work elucidates the psychological and neural processes that underlie how animals react to discrete cues in different contexts and has the potential to guide new treatments for substance use disorder.

## Methods

### Apparatus

Behavioural training and testing used equipment and software from Med-Associates Inc. (St. Albans, VT, USA). We used 12 conditioning chambers (ENV-009A) with stainless steel floors (ENV-009A-GF), each in a fan-ventilated (84−88 dB), sound-attenuating, melamine cubicle (53.6 × 68.2 × 62.8 cm). The right wall featured a fluid port (17.5 cm from rear wall, 9 cm from front door) that contained two wells (ENV-200R3AM). Fluid delivery into one well occurred through a 20 ml syringe attached to a pump (PHM-100, 3.33 rpm) located outside the cubicle. Fluid port-entries were measured with an infrared beam (ENV-205M) and recorded to a computer using Med PC-IV software, which also controlled fluid delivery and stimulus presentations. The upper left wall featured a clicker stimulus (ENV-135M, 8 dB above background), a continuous white noise stimulus generator (ENV-225SM, 8 dB above background), and a white house-light (ENV-215M).

### Solutions and reagents

Odours were prepared by adding lemon oil (SAFC Supply Solutions, St-Louis, MO, USA, #W262528) or benzaldehyde (almond odour, OMEGA Chemical Company Inc., Levis, QC, Canada, #B37-50) to tap water (10%, v/v). Alcohol (15 % ethanol, v/v) was prepared every week by diluting 95% ethanol in tap water (room temperature). Eticlopride (C_17_H_25_ClN_2_O_3_^.^ HCl, Sigma Aldrich, #E101) and SCH23390 (C_17_H_18_ClNO^.^ HCl, Sigma Aldrich, #D054) were dissolved in sterile 0.9% saline to make separate 10 μg per ml solutions. Clozapine-*n*-oxide (Tocris #4936 or NIMH C-929) for systemic administration was dissolved in 5% dimethyl sulfoxide and 95% sterile 0.9% saline to make 10 or 20 mg per ml concentrations. Clozapine (AdooQ, #A10236-500) was dissolved in 5% dimethyl sulfoxide and 95% sterile 0.9% saline to make a 0.1 mg per ml solution. Clozapine-*n*-oxide (Abcam, #ab141704) was dissolved in sterile 0.9% saline (0.3 mM for intracerebral microinfusions) or artificial cerebrospinal fluid (ACSF) (1 μM for in vitro electrophysiology). Viral vectors were bought from the University of North Carolina Vector Core [AAV8-hSyn-DIO-hM4D(Gi)-mCherry (titre 5.3 or 4.6 × 10^12^), AAV8-hSyn-DIO-hM3D(Gq)-mCherry (titre 5.9 × 10^12^)] or Addgene [AAV8-hSyn-DIO-hM4D(Gi)-mCherry (titre 2.06 × 10^12^), AAV8-hSyn-DIO-mCherry (titre 2.1 × 10^13^)].

### Subjects

Twenty-five wild-type male, Long-Evans (220−275 g on arrival, INVIGO), 20 wild-type outbred (4 female, 16 male) Long-Evans (bred in-house) and 94 outbred male, Long-Evans, TH::Cre^+/−^ rats (bred in-house) were single-housed in cages with bedding and a nylabone^TM^ chew toy in a vivarium (21 ± 2 °C, 40−50% humidity) maintained on a 12 h light−dark cycle (lights ON at 07:00, all procedures occurred in the light phase). Rats had unrestricted access to chow (Charles River Rodent Diet, #5075) and water. Founder TH::Cre rats were generously provided by Dr. Karl Deisseroth^58^. Procedures were approved by the Animal Research Ethics Committee at Concordia University and complied with guidelines from the Canadian Council on Animal Care.

In total, 12 rats failed to acquire Pavlovian conditioning (mean of <5 CS port-entries per session across last two sessions), 9 had missed cannula placements, 1 had a brain lesion from infection in the target region, 2 experienced a programming error, and 1 had the house-light burn out at test. Data from these rats were excluded.

### Surgery

Anesthetized (isoflurane, 5% induction, 2−3% maintenance) rats were secured in a stereotaxic frame and administered atropine (0.1 ml per kg) subcutaneously (s.c.). Bilateral, VTA microinfusions of 1 μl (0.1 μl per min, 10 min diffusion) of AAV8-hSyn-DIO-hM4D(Gi)-mCherry, AAV8-hSyn-DIO-mCherry, or AAV8-hSyn-DIO-hM3D(Gq)-mCherry viral vectors were made through a 26-gauge injector connected with PE20 tubing to a Hamilton microinjection syringe on a Harvard Apparatus, Pump 11 Elite. Ventral tegmental area coordinates (in mm) from bregma were: AP -5.5, ML ± 1.84 (with 10° angle), DV −8.33.

Where appropriate, 26-gauge bilateral guide cannulae (PlasticsOne, C315G-SPC) were implanted 3 mm dorsal to the microinjection site using the following coordinates (in mm) at a 10° angle: NAc core AP +1.2, ML ±3.23, DV −7.11, and NAc shell AP +1.68, ML ±2.23, −7.35. After surgery, rats received buprenorphine (0.1 mg per kg, s.c.) and ≥7 days to recover.

### Home-cage alcohol exposure

Rats were acclimated to the taste and pharmacological effects of ethanol^51,81^ across 12, 24-h sessions of access to 15% ethanol (alcohol) every other day in the home-cage, with water always available. Alcohol was provided in 100 ml graduated cylinders and water in 500 ml bottles. Containers were weighed before and after every 24-h session, and their positions in the lid of the home-cage were switched in every session. Fluid loss was estimated from control containers placed onto two empty cages. Rats that drank less than 1 g per kg of alcohol on session 6 were given two sessions of access to 5% ethanol and then returned to 15% ethanol.

Experiment 4 used 10% sucrose instead of alcohol. Rats were acclimated to 10% sucrose in the home-cage across two, consecutive 24-h sessions as described above.

### Pavlovian conditioning with context alternation and tests for CS-triggered alcohol-seeking

On the last day of home-cage alcohol exposure, rats were individually handled in the behavioural testing room. On the 2 subsequent days, they were habituated (20 min) to context 1 and, 24 h later, to context 2 in the conditioning chambers. Context 1 included black walls, a clear polycarbonate floor, and a lemon odour. Context 2 included clear Plexiglas walls, a wire-mesh floor, and an almond odour. Odours (three sprays) were applied to a petri dish placed in the waste-pan under the chamber floor.

Rats were assigned to one of the two contexts for Pavlovian conditioning (alcohol context), while the second context served as the neutral context. Discrete stimuli were a 10-s, continuous white noise or 10-s clicker (5 Hz). One stimulus (CS) was paired with alcohol delivery in the alcohol context and the other (neutral stimulus, NS) was presented without alcohol in the neutral context. The purpose of the NS was to equate the acoustical salience of both contexts. Rats were counterbalanced across contexts, stimulus identity, and session order such that there were no differences in home-cage alcohol consumption. Rats then received one training session per day (73.5 min) that alternated between each context until 12 sessions of Pavlovian conditioning in the alcohol context and 12 sessions of exposure to the NS in the neutral context had occurred.

During training sessions, rats received 15 stimulus presentations (either CS or NS as per the appropriate context) with intervals of 140, 260, or 380 s between trials [mean inter-trial interval (ITI) = 260 s, based on pilot studies]. In the alcohol context, CS presentations co-terminated with 6 s of syringe pump operation to deliver 0.2 ml of alcohol into the fluid port. After each session in the alcohol context fluid ports were examined to ensure that the alcohol had been consumed and noted as follows: 1 (dry, 0 ml), 2 (slightly wet, <0.2 ml), 3 (wet, 0.2 ml), 4 (very wet, >0.2 ml). In the neutral context, NS presentations also co-terminated with 6 s of syringe pump operation, but no alcohol was delivered.

The protocol described above was conducted identically in all experiments, with two exceptions. First, in experiment 1, we examined if having an NS present or absent from sessions in the neutral context would impact CS-triggered alcohol-seeking at test. There were no differences in acquisition or at test between rats that were trained with or without an NS, so all subsequent experiments included an NS in the neutral context. Second, in experiment 4, 10% sucrose was used instead of alcohol.

At 24 h after the last training session, we tested responding triggered by the CS alone. In all tests, the CS was presented as during Pavlovian conditioning sessions and syringe pumps were activated, but no alcohol was delivered. The order of the test context and all treatment conditions were counterbalanced in all experiments according to a Latin-square design.

Specific conditions for each experiment included in the main manuscript are described below. Methods for experiments included in the Supplementary material are provided in the corresponding figure captions.

### Experiment 1a: Is the impact of context on CS-triggered alcohol-seeking transient or persistent?

Following training as described above, we tested CS port-entries in the alcohol context and in the neutral context in male, Long-Evans rats (*n* = 22). Rats then had two re-training sessions, one per day in each context, prior to a second test that occurred in the opposite context.

Starting 24 h after the second test, we conducted repeated daily test sessions to examine if the elevation of CS port-entries in an alcohol context was transient or persistent. All rats were presented with the CS as during training, without alcohol, in the alcohol and neutral contexts on alternating days for a total of eight sessions (four per context).

After the last repeated test, at which point CS port-entries had extinguished to similar levels in both contexts, we examined the impact of context on CS-triggered alcohol-seeking in a relapse model. All rats received a reinstatement test in the opposite context from the one in which the last of the repeated test sessions had occurred (test in neutral context, *n* = 12; test in alcohol context *n* = 10). At test, a 0.2 ml drop of alcohol was dispensed over 6 s into the fluid port 30 s after placement in the chamber. A second 0.2 ml drop of alcohol was delivered during the last 6 s of the first CS, with no more alcohol delivered for the remainder of the test. This prime served as a reminder of the taste and smell of alcohol.

### Experiment 1b: Is dopamine required for CS-triggered alcohol-seeking in a neutral context?

A subset of rats from experiment 1a (*n* = 11) received three Pavlovian conditioning sessions alternating with three sessions of exposure to the NS in the neutral context. All rats received two 1 ml per kg s.c. saline habituation injections, 15 min before each of the last two training sessions, one in each context. Next, three tests were conducted in the neutral context. Tests started 15 min after systemic (s.c.) administration of vehicle, the dopamine D2-like receptor antagonist eticlopride (10 μg per kg), or the D1-like receptor antagonist SCH23390 (10 μg per kg)^26,51^. Tests were separated by a single Pavlovian conditioning session in the alcohol context and followed a within-subjects design.

### Experiment 2: Effect of chemogenetic inhibition of VTA dopamine neurons on CS-triggered alcohol-seeking in a neutral context

TH::Cre rats expressing hM4Di in the VTA (*n* = 12) received two 1 ml per kg saline injections (intraperitoneal, i.p.), one in each context, 30 min before each of the last two training sessions. Next, three tests for CS-triggered alcohol-seeking in the neutral context were conducted after vehicle, CNO at 10 mg per kg, or CNO at 20 mg per kg (i.p.) injection^30,53^. Tests were separated by four retraining sessions, alternating between the alcohol and neutral contexts (two sessions per context).

### Experiment 3: Potential off-target effects of CNO and its parent compound clozapine

TH::Cre rats expressing mCherry in the VTA (*n* = 13) received two 1 ml per kg saline habituation injections (i.p.), one in each context, 30 min before each of the last two training sessions. Next, three tests for CS-triggered alcohol-seeking in the neutral context were conducted 30 min after vehicle, 10 mg per kg CNO (the lowest effective dose to reduce CS port-entries in experiment 2), or 0.1 mg per kg clozapine (a dose that would be produced by the reverse metabolism of 10 mg per kg CNO) (i.p.). Tests were separated by a retraining session in the alcohol context and in the neutral context.

### Experiment 4: Effect of chemogenetic inhibition of VTA dopamine neurons on CS-triggered sucrose-seeking in a neutral context

Detailed methods are included in the caption for Supplementary Fig. 7.

### Experiment 5: Assessing off-target effects of CNO microinfusion in the nucleus accumbens core and shell

Detailed methods are included in the caption for Supplementary Fig. 8.

### Experiment 6: Chemogenetic inhibition of dopaminergic VTA-to-NAc core circuit

TH::Cre rats expressing hM4Di in the VTA (*n* = 8) received a saline habituation microinfusion (0.15 μl over 1 min) into the NAc core 5−15 min before a training session in the alcohol and neutral contexts. At 24 h after the last training session, the first of four tests for CS-triggered alcohol-seeking was conducted. A similar number of rats was tested in the alcohol or neutral context after receiving a vehicle or CNO (3 mM)^56,57^ microinfusion (0.3 μl over 1 min) 5−15 min before the test^53,55,56^. One retraining session in the alcohol context and one in the neutral context separated each of the four tests.

### Experiment 7: Chemogenetic inhibition of dopaminergic VTA-to-NAc shell circuit

TH::Cre rats expressing hM4Di in the VTA (*n* = 11) received a saline habituation microinfusion (0.15 μl over 1 min) into the NAc shell before a training session in the alcohol and neutral context. At 24 h after the last training session, the first of four tests for CS-triggered alcohol-seeking was conducted. A similar number of rats was tested in the alcohol or neutral context after receiving a vehicle or CNO (3 mM)^56,57^ microinfusion (0.3 μl over 1 min) 5−15 min before the test session. One retraining session in the alcohol context and one in the neutral context separated each of the four tests.

### Experiment 8: Viral transduction efficiency and selectivity for TH+ neurons of hSyn-DIO-hM4Di-mCherry in TH::Cre rats

TH::Cre rats (*n* = 4) were deeply anesthetized with euthanyl^TM^ (sodium pentabarbitol, 240 mg per kg, i.p.) and perfused with phosphate-buffered saline (PBS, 0.02 M, pH 7.2) and 4% paraformaldehyde in 0.02 M PBS (150 ml, pH 7.2). Brains were immediately removed, cryoprotected in a 4% paraformaldehyde, 30% sucrose solution (50 ml, 2−3 days), and then stored at −80 °C. Brains were sectioned (40-μm thick) using a cryostat (−20 °C) and thaw-mounted onto slides that were stored at −20 °C.

For immunohistochemistry, slides were removed from the −20 °C freezer, covered and allowed to dry, in a fumehood overnight. The entire slide, excluding the label, was then outlined with an ImmEdge^TM^ hydrophobic barrier pen (Vector Labs, #H-4000) and then washed twice by pipetting 500 μl of 0.01 M PBS onto the slide for 1 min. After the second wash, 500 μl of 10% normal donkey serum (NDS, Sigma Aldrich, #D9663) in 0.01 M PBS plus 0.3% Triton X-100 (PBST) was applied to the slide for 30 min. After a 1 min wash in 0.01 M PBS, 500 μl of mouse anti-mCherry (1:1000, Abcam, #ab125096) and rabbit anti-TH (1:100, EMD Millipore, #ab152) in 10% NDS PBST was applied to the slides and left to incubate for 48 h at room temperature. Slides were then washed three times with 500 μl of 0.01 M PBS for 5 min. Then, 500 μl of donkey anti-mouse IgG (H + L) alexa 594 (1:200, Jackson ImmunoResearch labs, #715-585-150) and donkey anti-rabbit IgG (H + L) alexa 488 (1:200, Jackson ImmunoResearch Labs, #711-545-152) in 0.01 M PBS was applied to the slides and left to incubate for 24 h at room temperature. Slides were left to dry covered at room temperature for 2 h and then cover-slipped with vectasheild^TM^ (Vector Labs, #H-1200) and imaged immediately or stored at 4 °C until imaging.

To verify that hM4Di was selectively expressed in TH^+^ cells, six coronal sections spanning 1.2 mm through the VTA from each of four rats were imaged using a Nikon laser scanning C2 system (Nikon NIS Elements; Fiji v2.0). Sections were surveyed at ×4 magnification using a 594 nm excitation filter cube epifluorescence to identify the coronal section with the most expansive mCherry fluorescence. This coronal section was then imaged at ×4 magnification with 405, 488, and 561 nm lasers to capture a three-channel (DAPI, mCherry, TH) image of the entire section. Then, 2−4 images per hemisphere were taken at ×40 magnification to again capture a three-channel image (~300 × 300 μm) for co-localization analyses.

### Experiment 9: In vitro intracellular recordings of NAc core medium spiny neurons innervated by VTA dopamine terminals expressing designer receptors

Male, TH::Cre rats (*n* = 10) received stereotaxic surgery to deliver VTA microinfusions of 1 μl of AAV8-hSyn-DIO-hM4D(Gi)-mCherry (*n* = 5) or AAV8-hSyn-DIO-hM3D(Gq)-mCherry (*n* = 5). At 4−6 weeks later, rats were anaesthetized with isoflurane and decapitated. Brains were rapidly extracted and submerged in an ice-cold HEPES-based ACSF solution containing (in mM): 92 NaCl, 2.5 KCl, 1.2 NaH_2_PO_4_, 20 HEPES, 30 NaHCO_3_, 25 glucose, 5 sodium ascorbate, 2 thiourea, 3 sodium pyruvate, 12 *N*-acetyl-l-cysteine (NAC), 10 MgSO_4_, and 0.5 CaCl_2_ (pH adjusted to ≈7.3−7.4 using 10 M NaOH) saturated with 95% O_2_ per 5% CO_2_. Coronal slices (300 μm) containing the NAc were obtained using a vibratome (Leica, VT1200) and transferred to a warm (34 ˚C), high-choline incubation solution containing (in mM): 92 choline chloride, 2.5 KCl, 1.2 NaH_2_PO_4_, 30 NaHCO_3_, 20 HEPES, 25 glucose, 5 sodium ascorbate, 2 thiourea, 3 sodium pyruvate, 12 NAC, 10 MgSO_4_, and 0.5 CaCl_2_, where they recovered for 12 min. Subsequently, slices were incubated at room temperature, in a normal ACSF solution containing (in mM): 124 NaCl, 5 KCl, 1.25 NaH_2_PO_4_, 2 MgSO_4_, 26 NaHCO_3_, 2 CaCl_2_, and 10 dextrose and were allowed to recover for a minimum of 1 h prior to experiments. Once transferred into the recording chamber, slices were perfused with normal ACSF at 2 ml per min and were visualized using an upright fluorescence microscope with a ×40 water-immersion objective, differential interference contrast optics (Olympus, BX51WI), and XM-10 monochrome camera for viewing (Olympus, CellSens v1.8). mCherry fluorescence in the NAc core was verified at ×4 and ×40 magnification prior to recordings (see Supplementary Fig. 11).

Whole-cell patch-clamp pipettes made from borosilicate glass (1.0 mm OD, 3−5 MΩ) were filled with a recording solution containing (in mM): 140 K-gluconate, 5 NaCl, 2 MgCl_2_, 10 HEPES, 0.5 EGTA, 2 ATP-tris, 0.4 GTP-tris (pH adjusted to 7.25 using KOH, 270−280 mOsm) and were lowered onto visually identified neurons in the NAc core. Tight seals were obtained (1.3−6.6 GΩ), and cells were allowed to stabilize in whole-cell configuration for 10 min prior to recordings. Recordings were obtained using a Multiclamp 700 B amplifier (Molecular Devices), digitized (Digidata 1440A, Molecular Devices), and were stored using pClamp 10.3 software (Molecular Devices). Access resistance was 19.9 ± 2.2 MΩ, and series resistance was uncompensated. All cells recorded had a resting membrane potential below −65 mV. Cellular input resistance, membrane capacitance, and access resistance were monitored during each recording condition.

Cells were initially selected based on visual criteria; MSNs and GABAergic interneurons possess smaller soma in comparison to cholinergic interneurons (8−20 μm vs. 20−50 μm^59,82^) and any cells with soma > 30 μm were not recorded from. GABAergic interneurons and medium spiny projection (MSN) neurons of the accumbens core were differentiated electrophysiologically by injecting 500 ms hyperpolarizing and depolarizing current steps between −100 and 100 pA in 10 pA intervals from the holding potential of −70 mV. Peak input resistance was measured at the largest voltage change in response to a −100 pA pulse, and steady-state input resistance was assessed just prior to the end of the current step. Action potential properties were measured from the first action potential evoked in response to positive current injection.

Synaptic responses were evoked using a bipolar stimulating electrode made from two tungsten electrodes (≈1 MΩ, FHC Inc.) placed approximately 30 μm from the recording electrode. Evoked AMPA-receptor-mediated EPCSs were recorded at −70 mV, near the resting membrane potential of MSNs of the NAc core^83^ using constant current stimulation pulses. For each cell, at least ten consecutive synaptic responses free from artifacts or action potentials were averaged for each phase of the recordings. Dopamine release from VTA terminals to the NAc core is under tonic inhibition from aspiny GABAergic interneurons, and dopamine can also modify inhibition in the accumbens^83,84^. Because GABA neurotransmission can alter the excitability of MSNs, we included picrotoxin (50 μM) in the ACSF to block GABA_A_-mediated inhibition and better assess the effects of CNO on VTA inputs to medium spiny neurons. Recordings were obtained before and after 5 min (hM4Di) or 10 min (hM3Dq) application of 1 μM CNO, and were also obtained after 20 min washout of CNO in the continued presence of picrotoxin. The amplitudes of averaged synaptic currents were measured using Clampfit 8.2 software (Molecular Devices) and normalized to the amplitude of responses recorded prior to CNO application.

### Experiment 10: Assessing the diffusion of CNO from the NAc core to the NAc shell following microinfusion

Detailed methods are included in the caption for Supplementary Fig. 12.

### Histology and imaging

Rats from experiments 2−7 were deeply anesthetized with euthanyl^TM^ (sodium pentabarbitol, 240 mg per kg, i. p.) and perfused with PBS (0.02 M, 250 ml, pH 7.2) and 4% paraformaldehyde in 0.02 M PBS (150 ml, pH 7.2). Brains were immediately removed, cryoprotected in a 4% paraformaldehyde 30% sucrose solution (50 ml, ~2−3 days), and then stored at −80 °C until they were sectioned (40-μm thick) using a cryostat at −20 °C. Nissl staining was conducted to assess histological placements of injector tips in the NAc core and shell. Unamplified mCherry signal was used to verify successful designer receptor expression by letting slides dry for 24 h after removal from the −20 °C freezer and then coverslipping them with vectasheild^TM^. We assessed mCherry fluorescence using a Leica DM4000B epifluorescence microscope at ×5 and ×10 magnification.

### Analyses and statistics

Statistical analyses were conducted with SigmaPlot^TM^ v12 or SPSS^TM^ v20 and graphs were made with SigmaPlot^TM^ v12.

Dependent variables: During home-cage alcohol consumption, we measured grams of ethanol consumed per kilograms of body weight (g per kg). In Pavlovian conditioning and test sessions, we measured the number of: port-entries per session (total port-entries), port-entries during the 10 s CS (CS port-entries), port-entries made between CS offset and the next CS onset (140, 260, or 380 s; NonCS port-entries), port-entries in the 10 s preceding the CS (PreCS port-entries), and CS port-entries minus PreCS port-entries (Normalized CS port-entries).

Data from all experiments were first analysed using repeated-measures (RM) ANOVA with the factor Session and the factors Context (Alcohol, Neutral), Interval (PreCS, CS) and Treatment (Vehicle, CNO), or Treatment (Vehicle, CNO, Clozapine), or CNO Dose (Vehicle, 10 mg per kg, 20 mg per kg), or Dopamine Antagonist (Vehicle, Eticlopride, SCH23390) as determined by the experiment. A Huynh−Feldt correction was applied when sphericity was violated in these analyses. Significant interactions were decomposed with follow-up two-tailed Bonferroni-corrected *t* tests or Newman−Keuls post hoc tests. The electrophysiology data met the requirements for normality as indicated by the Kolmogorov−Smirnov test with Lilliefors’ correction. All analyses used an alpha level of *p* = 0.05.

### Reporting summary

Further information on experimental design is available in the Nature Research Reporting Summary linked to this paper.

## Supplementary information

Supplementary Information

Description of Additional Supplementary Files

Supplementary Movie 1

Reporting Summary

## Data Availability

Raw data for all experiments are available as a supplementary source data file. Source data are provided with this paper.

## Source data

Source Data
