# Supplementary material for: Dissociable mesolimbic dopamine circuits control responding triggered by alcohol-predictive discrete cues and contexts
Source: Nat Commun. 2020 Jul 28;11:3764. doi: 10.1038/s41467-020-17543-4 (PMC7534644; doi:10.1038/s41467-020-17543-4)
Supplement: Supplementary file 1 — Supplementary Information [file 41467_2020_17543_MOESM1_ESM.pdf]

Dissociable mesolimbic dopamine circuits control responding triggered by alcohol-predictive discrete cues and contexts

M. Valyear, I. Glovaci, A. Zaari, S. Lahlou, I. Trujillo-Pisanty,A. Chapman,

N. Chaudhri

Group	Alcohol Context	Neutral Context	CS	NS	# of Rats by experiment						
					1	2	3	4	5	6	7
CS/NS	1	2	White Noise	Clicker	3	4	1	5	4	0	4
	1	2	Clicker	White Noise	2	1	4	4	4	3	1
	2	1	White Noise	Clicker	3	4	5	4	3	3	4
	2	1	Clicker	White Noise	3	3	3	5	4	2	2
CS/NoNS	1	2	White Noise	NA	2						
	1	2	Clicker	NA	3						
	2	1	White Noise	NA	3						
	2	1	Clicker	NA	3						
Total n					22	12	13	18	15	8	11

Supplementary Table 1 | Breakdown of the number of rats from each experiment that received the different combinations of discrete, auditory stimuli used as the conditioned stimulus (CS) or neutral stimulus (NS) in each of the 2 context configurations used as either the alcohol context or neutral context. Experiment 4 used 10% sucrose solution instead of 15% ethanol. Context 1 had dark walls, a smooth polycarbonate floor, and a lemon odour. Context 2 had clear walls, a wire-mesh floor, and an almond odour.

Experiment	n	Figure(s)	Description
1a	22	1, S1a, S2a, S3a, S4a, S5	Is the impact of context on CS-triggered alcohol-seeking transient or persistent?
1b	11	2b	Is dopamine required for CS-triggered alcohol-seeking in a neutral context?
2	12	2c, S1b, S2b, S3b, S4b, S6a	Effect of chemogenetic inhibition of VTA dopamine neurons on CS-triggered alcohol-seeking in a neutral context.
3	13	2d, S1c, S2c, S3c, S4c, S6b	Potential off-target effects of CNO and its parent compound clozapine.
4	18	S7	Effect of chemogenetic inhibition of VTA dopamine neurons on CS-triggered sucrose-seeking in a neutral context.
5	15	S8	Assessing off-target effects of CNO microinfusion in the nucleus accumbens core and shell.
6	8	31, 3b, 3c, 3d, S1d, S2d, S3d, S4d, S6c, S9	Chemogenetic inhibition of dopaminergic VTA-to-NAc core circuit.
7	11	4a, 4b, 4c, 4d, S1e, S2e, S3e, S4e, S6d, S10	Chemogenetic inhibition of dopaminergic VTA-to-NAc shell circuit.
8	4	4a-d, S9, S10	Viral transduction efficiency and selectivity for TH+ neurons of hSyn-DIO-hM4Di-mCherry in TH::Cre rats.
9	10	4e-j, S11	In vitro intracellular recordings of NAc core medium spiny neurons innervated by VTA dopamine terminals expressing designer receptors.
10	1	S12	Assessing the diffusion of CNO from the NAc core to the NAc shell following microinfusion.

Supplementary Table 2│ For use when referring to methods and supplementary figure captions. Experiments are listed by number, and the sample size (n), relevant Figure numbers, and brief descriptions are provided.

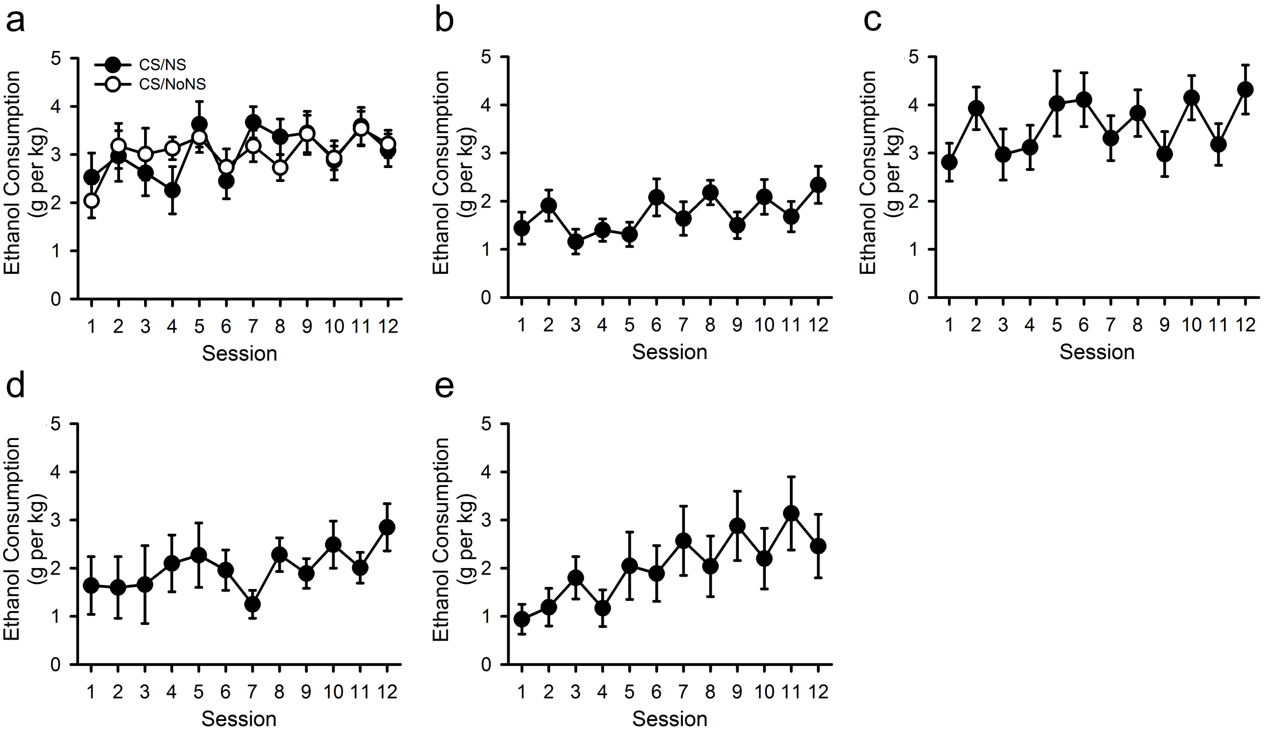

Supplementary Fig. 1│ Consumption of 15% ethanol increased over sessions of intermittent access to alcohol in the home-cage. Rats received 24 h access to a 15% ethanol solution in their home-cage every other day for a total of twelve sessions. Water and standard rat chow were continuously available. **a** Ethanol consumption in wild-type rats increased across sessions, but did not differ between separate groups of rats that either did or did not receive a neutral auditory stimulus (NS) during sessions in the neutral context in Experiment 1 [Session, $F_{(11, 220)}$=3.875, p<.001; Group, $F_{(1, 20)}$<.001, p=.993; Session x Group $F_{(11, 220)}$=1.106, p=.358]. Ethanol consumption increased across sessions in TH::Cre$^{+/-}$ rats in: **b** Experiment 2, [$F_{(11, 132)}$=3.799, p<.001]. **c** Experiment 3, [$F_{(11, 154)}$=4.139, p<.001]. **d** Experiment 6, [$F_{(11, 77)}$=2.257, p=.019]. **e** Experiment 7, [$F_{(11, 110)}$=7.140, p<.001]. Averaged data are mean ± s.e.m. Data were analyzed using RM ANOVA. All statistical tests were two-sided. Raw data are available as a supplementary source data file.

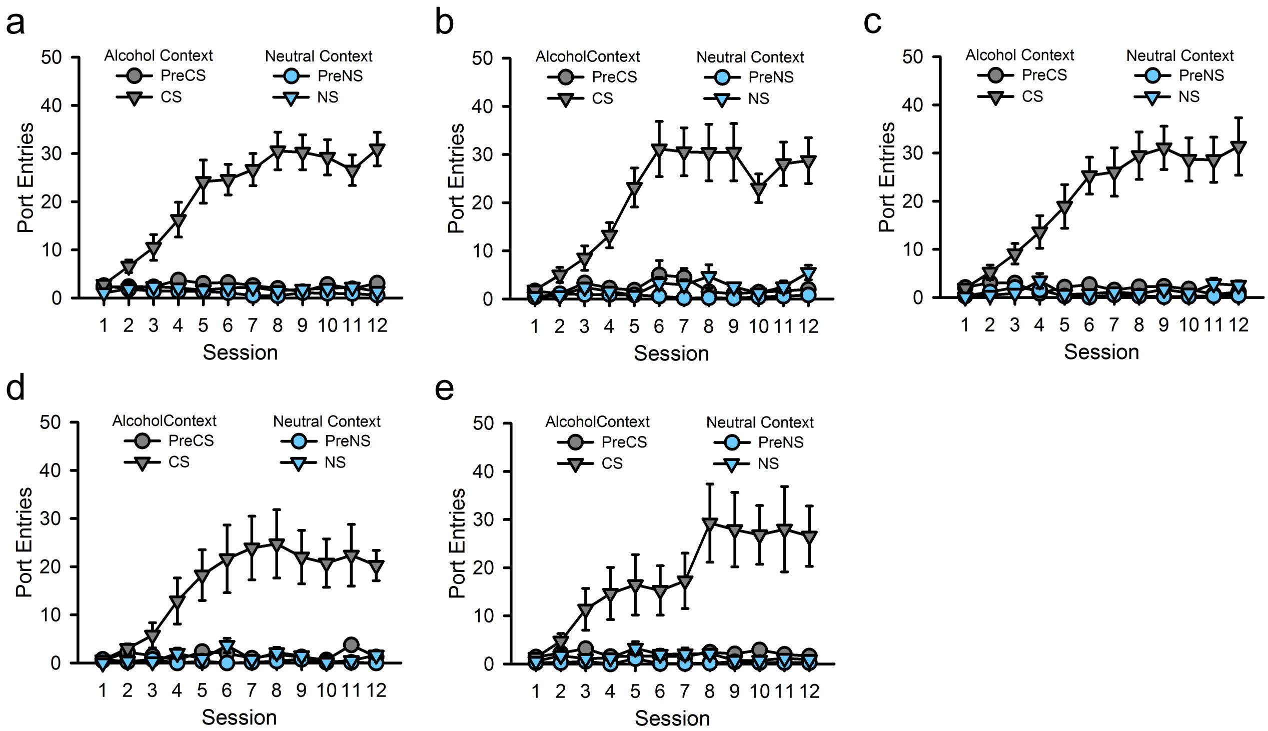

Supplementary Fig. 2 | Acquisition of Pavlovian conditioning with context alternation. Rats received Pavlovian conditioning sessions every other day in a distinct alcohol context where a discrete, 10 s auditory conditioned stimulus (CS; 15 trials per session) was paired with 15% ethanol (alcohol; 0.2 ml per CS; 3.0 ml per session; variable inter trial interval = 260 s). On alternating days, rats were exposed to a different, neutral context where a distinct, 10 s neutral auditory stimulus (NS; 15 trials per session) was presented without alcohol. **a** In Experiment 1, while in the neutral context, half of the rats received presentations of an auditory stimulus that was distinct from their CS (referred to as the neutral stimulus, NS group), whereas the remainder did not (noNS group; **Supplementary Table 1**). The purpose of these 2 groups was to determine if the acoustical salience of the neutral context would impact CS port-entries at test. Since there were no main effects or interactions with group during acquisition or at test, the data reported here were collapsed across group. In all other experiments an NS was presented in the neutral context during training. CS-triggered port-entries increased into a plateau over Pavlovian conditioning sessions, whereas port-entries during the NS or during an equivalent period of time immediately before either stimulus (PreCS and PreNS) remained low. Experiment 1 [Session x Context x Interval, $F_{(11, 220)}$=17.37, p<.001]. **b** Experiment 2 [Session x Context x Interval $F_{(11, 121)}$=8.876, p<.001]. **c** Experiment 3 [Session x Context x Interval, $F_{(11, 132)}$=11.323, p<.001]. **d** Experiment 6 [Session x Context x Interval, $F_{(11, 77)}$=5.352, p<.001]. **e** Experiment 7 [Session x Context x Interval, $F_{(11, 110)}$=8.149, p<.001]. Averaged data are mean ± s.e.m. Data were analyzed using RM ANOVA. All statistical tests were two-sided. Raw data are available as a supplementary source data file.

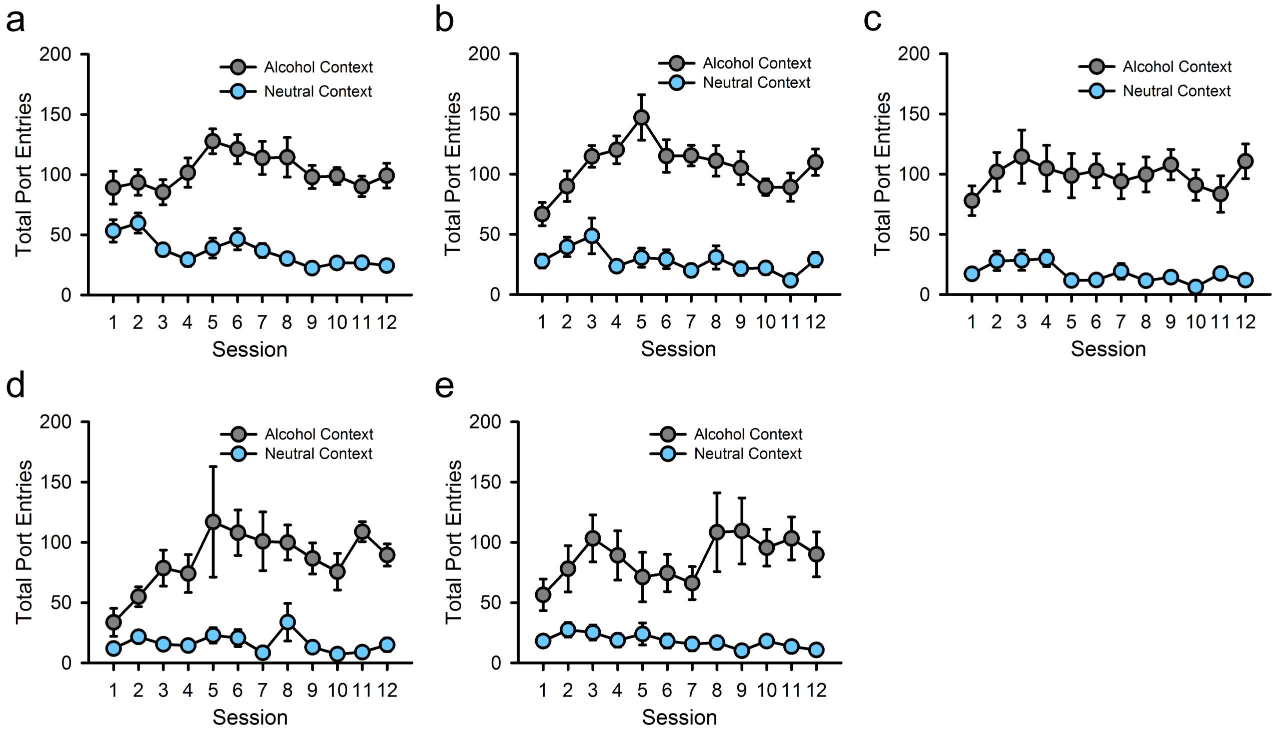

Supplementary Fig. 3│Total port-entries during the acquisition of Pavlovian conditioning with context alternation. Rats received Pavlovian conditioning sessions every other day in a distinct alcohol context where a discrete, 10 s auditory conditioned stimulus (CS; 15 trials per session) was paired with 15% ethanol (alcohol; 0.2 ml/CS; 3.0 ml per session; variable inter trial interval = 260 s). On alternating days, rats were exposed to a different, neutral context where a distinct, 10 s neutral auditory stimulus (NS; 15 trials/session) was presented without alcohol. The total number of port-entries made in each session was elevated in the alcohol context and was either low or decreased across session in the neutral context. **a** Experiment 1 [Context, $F_{(1, 20)}$=100.59, p<.001; Context x Session, $F_{(11, 220)}$=3.45, p<.001]. **b** Experiment 2 [Context, $F_{(1, 11)}$=129.409, p<.001; Context x Session, $F_{(11, 121)}$=3.054, p=.001]. **c** Experiment 3 [Context, $F_{(1, 12)}$=42.715, p<.001; Context x Session, $\mathrm{F}_{(11, 132)}$=13.480, p<.001]. **d** Experiment 6 [Context, $F_{(1, 7)}$=47.187, p<.001; Context x Session, $F_{(11, 77)}$=1.605, p=.114]. **e** Experiment 7 [Context, $F_{(1, 10)}$=25.726, p<.001; Context x Session, $F_{(11, 110)}$=2.235, p=.017]. Averaged data are mean ± s.e.m. Data were analyzed using RM ANOVA. All statistical tests were two-sided. Raw data are available as a supplementary source data file.

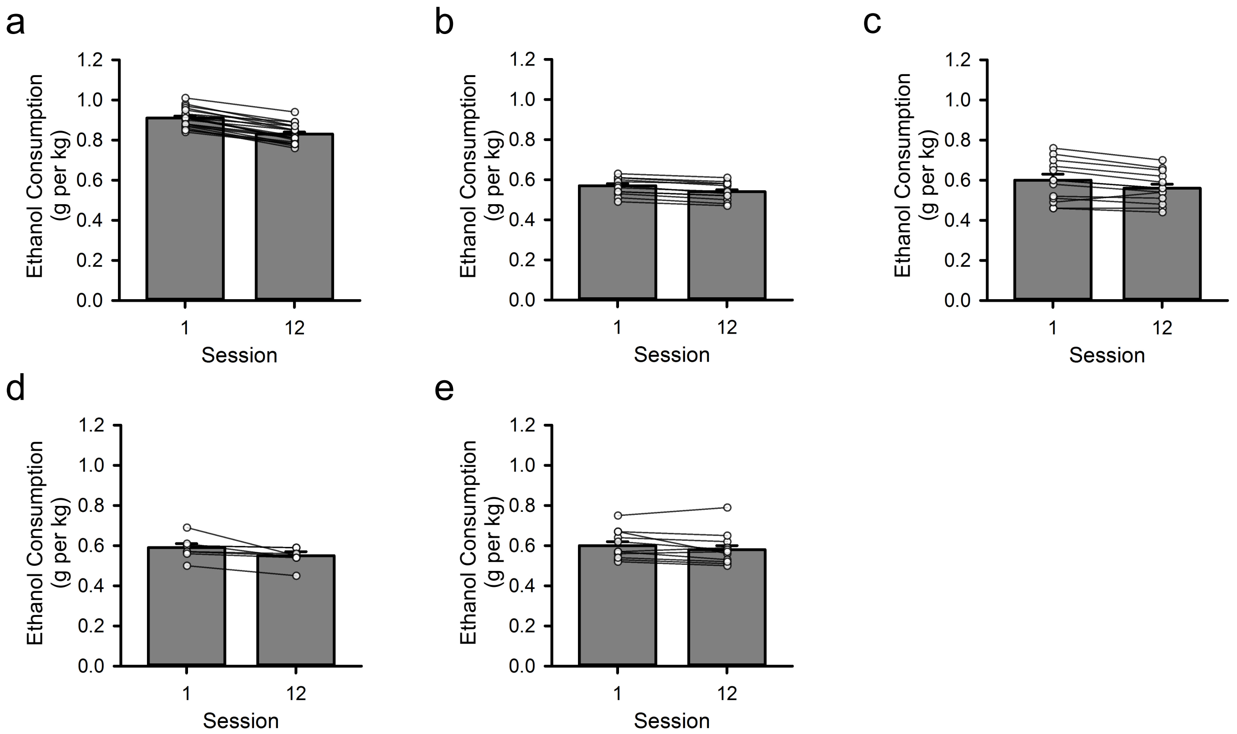

Supplementary Fig. 4│Ethanol consumption during Pavlovian conditioning with context alternation. Rats received Pavlovian conditioning sessions every other day in a distinct alcohol context where a discrete auditory conditioned stimulus (CS) was paired with 15% ethanol (.2 ml per trial; 3 ml per session). On alternating days, rats were exposed to a different, neutral context where a distinct, neutral auditory stimulus (NS) was presented without alcohol. The amount of ethanol consumed in grams over kilograms of bodyweight (g per kg) in the first and last session is shown here. Generally, ethanol consumption measured as a function of body weight was lower on the last Pavlovian conditioning session compared to the first session because the amount of ethanol delivered per session was fixed but rats continued to gain weight over time. **a** Experiment 1 [$t_{(21)}$=15.947, p<.001]. **b** Experiment 2 [$t_{(11)}$=10.557, p<.001]. **c** Experiment 3 [$t_{(12)}$=3.649, p=.003]. **d** Experiment 6 [$t_{(7)}$=2.565, p=.037]. **e** Experiment 7 [$t_{(10)}$=1.713, p=.118]. Averaged data are mean ± s.e.m. Data were analyzed using two-sided paired-samples t-tests. Raw data are available as a supplementary source data file.

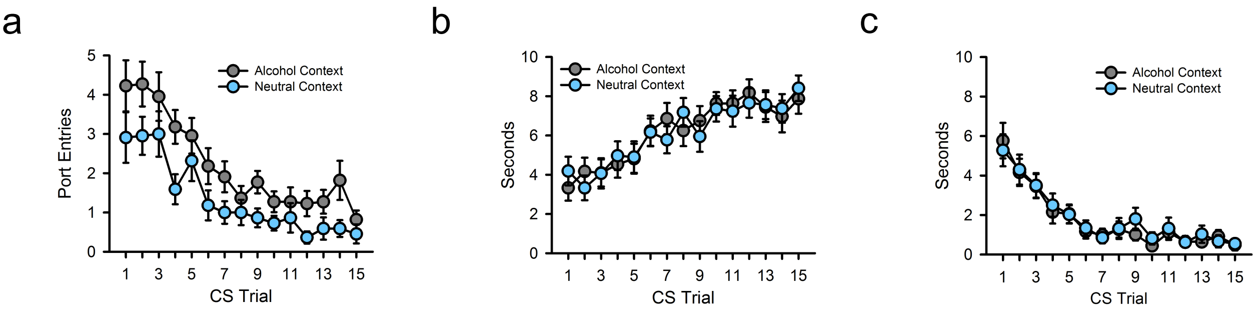

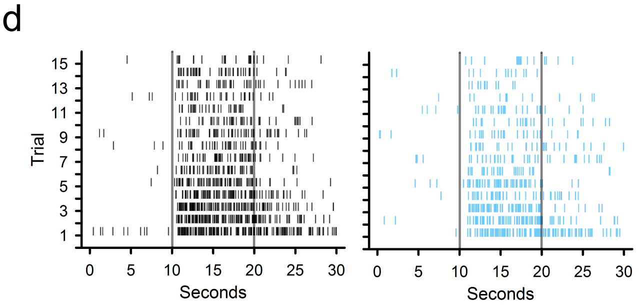

Supplementary Fig. 5│Microstructure of conditioned responding. Rats received Pavlovian conditioning sessions every other day in a distinct alcohol context where a discrete, 10 s auditory conditioned stimulus (CS; 15 trials per session) was paired with 15% ethanol (alcohol; 0.2 ml per CS; 3.0 ml per session; variable inter trial interval = 260 s). On alternating days, rats were exposed to a different, neutral context where a distinct, 10 s neutral auditory stimulus (NS; 15 trials per session) was presented without alcohol. Port-entries elicited by the CS (i.e., CS-triggered alcohol-seeking) were tested once each in the alcohol and neutral contexts by presenting the CS as during Pavlovian conditioning but without alcohol. We analyzed the frequency, latency, and duration of CS port-entries at test on a trial-by-trial basis. **a** The average number of CS port-entries (i.e. the total number of port-entries made during each CS) decreased across CS trials at test, but was elevated overall in the alcohol context [Context, $F_{(1, 20)}$=13.53, p<.001; Trial, $F_{(14, 280)}$=16.243, p=.001; Trial x Context, $F_{(14, 280)}$=.530, p=.915]. **b** The average latency to make a port-entry after CS onset (i.e. the number of seconds after CS onset before the first port-entry was made) increased across CS trials at test with no difference as a function of context [Trial, $F_{(14, 280)}$=12.329, p<.001; Context, $F_{(1, 20)}$=.006, p=.939; Context x Trial, $F_{(14, 280)}$= .488, p=.939]. **c** The average total duration of port-entries initiated during the CS (i.e. the total time spent in the port during port-entries initiated during the CS) decreased comparably across CS trials at test in both contexts [Trial, $F_{(14, 280)}$=19.79, p<.001; Context, $F_{(1, 20)}$=.169, p=.685; Context x Trial, $F_{(14, 280)}$= .279, p=.996]. **d** Raster plots showing every port-entry made during the PreCS (0-10 s), CS (10-20 s) and PostCS (20-30 s) intervals across all 15 CS trials by all rats at test in both the alcohol (black, left) and neutral (blue, right) contexts. Averaged data are mean ± s.e.m. Data were analyzed using RM ANOVA. All statistical tests were two-sided. Raw data are available as a supplementary source data file.

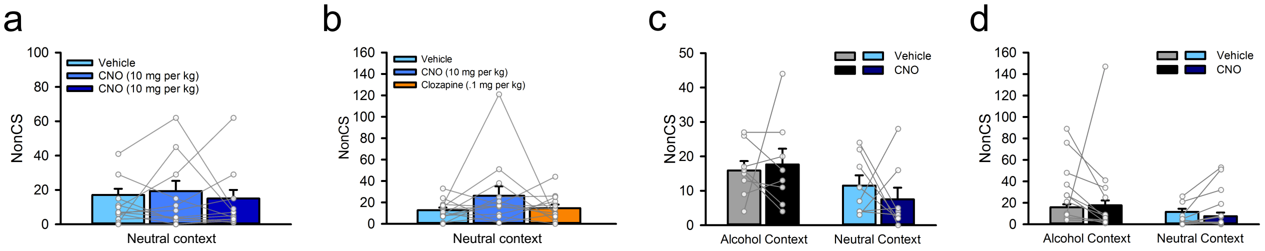

Supplementary Fig. 6│NonCS port-entries made during tests for CS-triggered alcohol-seeking. Port-entries made between CS trials (NonCS) are a measure of general port directed behavior at test. **a** Experiment 2: NonCS port-entries made in the neutral context by TH::Cre rats transfected with AAV8-hSyn-DIO-hM4Di-mCherry were similar across tests using difference doses of CNO [CNO dose, $F_{(2, 22)}$=.288, p=.752, $\eta_p{}^2$=.026]. **b** Experiment 3: NonCS port-entries made in the neutral context by TH::Cre rats transfected with AAV8-hSyn-DIO-mCherry were similar across tests using either vehicle, CNO or clozapine [Treatment, $F_{(2, 24)}$=1.678, p=.208, $\eta_p{}^2$=.123]. **c** Experiment 6: NonCS port-entries made by TH::Cre rats that were transfected with AAV8-hSyn-DIO-hM4Di-mCherry and received microinfusions of CNO or vehicle in the NAc core. NonCS port-entries were marginally elevated in the alcohol context, relative to the neutral context [Context, $F_{(1, 7)}$=4.307, p=.077, $\eta_p{}^2$=.381]. There was no effect of CNO on NonCS port-entries [Treatment, $F_{(1, 7)}$=.063, p=.809, $\eta_p{}^2$=.009] in either context [Context by Treatment, $F_{(1, 7)}$=.688, p=.434, $\eta_p{}^2$=.089]. **d** Experiment 7: NonCS port-entries made by TH::Cre rats that were transfected with AAV8-hSyn-DIO-hM4Di-mCherry and received microinfusions of CNO or vehicle in the NAc shell. NonCS port-entries were significantly elevated in the alcohol context relative to the neutral context [Context, $F_{(1, 10)}$=16.221, p=.002, $\eta_p{}^2$=.619]. There was no effect of CNO on NonCS port-entries [Treatment, $F_{(1, 10)}$=.03, p=.866, $\eta_p{}^2$=.003] in either context [Treatment x Context, $F_{(1, 10)}$=1.093, p=.32, $\eta_p{}^2$=.099]. Rats in Experiments 6 (**c**) and 7 (**d**) had 3 additional training sessions in each context between tests, and 4 total tests, which may account for why NonCS port-entries were elevated in these experiments, compared to Exp. 1, in which rats only had 1 additional retraining session between 2 tests. Averaged data are mean ± s.e.m. Data were analyzed using RM ANOVA. All statistical tests were two-sided. Raw data are available as a supplementary source data file.

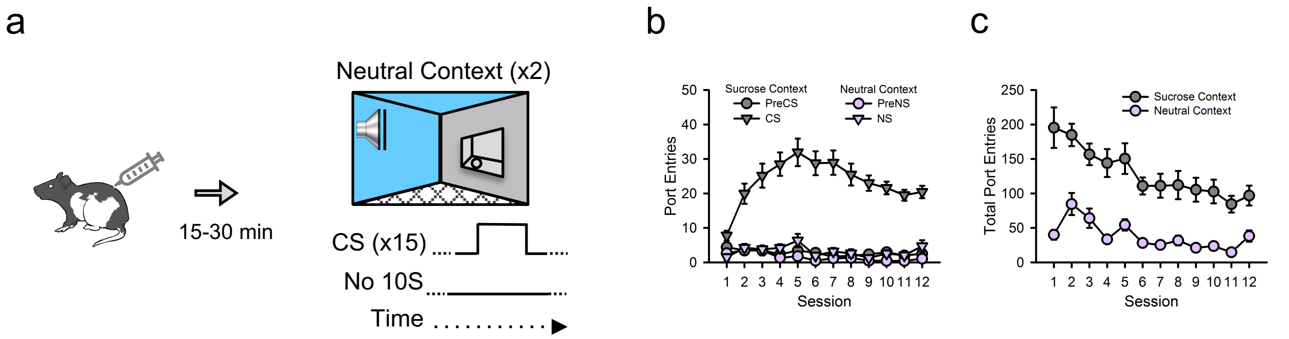

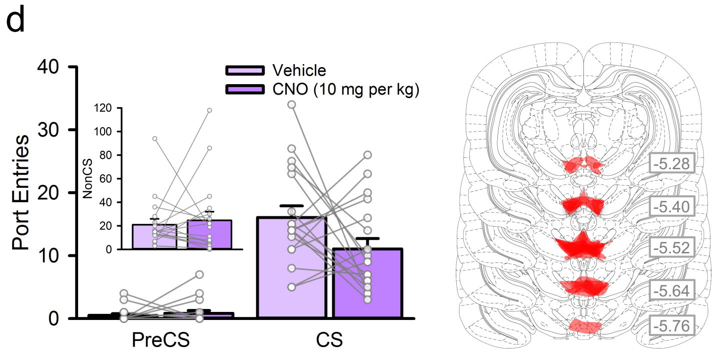

Supplementary Fig. 7│ Silencing VTA dopamine neurons did not attenuate CS-triggered sucrose-seeking. We examined the necessity of VTA dopamine neurons for CS-triggered sucrose-seeking. Rats (*n*=18) received Pavlovian conditioning sessions every other day in a distinct sucrose context where a discrete, 10 s auditory conditioned stimulus (CS; 15 trials per session) was paired with 10% sucrose (0.2 ml per CS; 3.0 ml per session; variable inter trial interval = 260 s). On alternating days, rats were exposed to a different, neutral context where a distinct, 10 s neutral auditory stimulus (NS; 15 trials per session) was presented without sucrose. All rats received two 1 ml per kg saline habituation injections (i.p.), one in each context, 30 min before each of the last two training sessions. Two tests for CS-triggered sucrose-seeking in the neutral context were then conducted 30 min after vehicle or our lowest effective does of 10 mg per kg CNO (i.p.). Tests were separated by three retraining sessions, two in the sucrose context intervened by one in the neutral context. **a** All rats were tested for CS-triggered sucrose-seeking in a neutral context after chemogenetic silencing of VTA dopamine neurons. **b** CS port-entries increased into a plateau across conditioning sessions whereas port-entries during the NS or preceding either interval (PreCS, PreNS) remained stably low [Session x Context x Interval, $F_{(11, 187)}$=5.140, p<.001]. **c** Total port-entries waned across sessions but decreased to a lower level in the neutral context [Context, $F_{(1, 17)}$=64.549, p<.001; Context x Session, $F_{(11, 187)}$=3.215, p<.001]. **d** At test, CS port-entries in the neutral context were elevated relative to PreCS port-entries and were similar following treatment with vehicle or our lowest effective does of 10 mg per kg CNO [Interval x Treatment, $F_{(1, 17)}$=3.691, p=.072; η_p^2=.178]. The inset graph shows NonCS port-entries, which were similar across both tests [Treatment, $F_{(1, 17)}$=.173, p=.682, η_p^2=.141]. Histology sections (right) depict maximal mCherry expression in the midbrain for every rat. Averaged data are mean ± s.e.m. Data were analyzed using RM ANOVA. All statistical tests were two-sided. Raw data are available as a supplementary source data file.

a

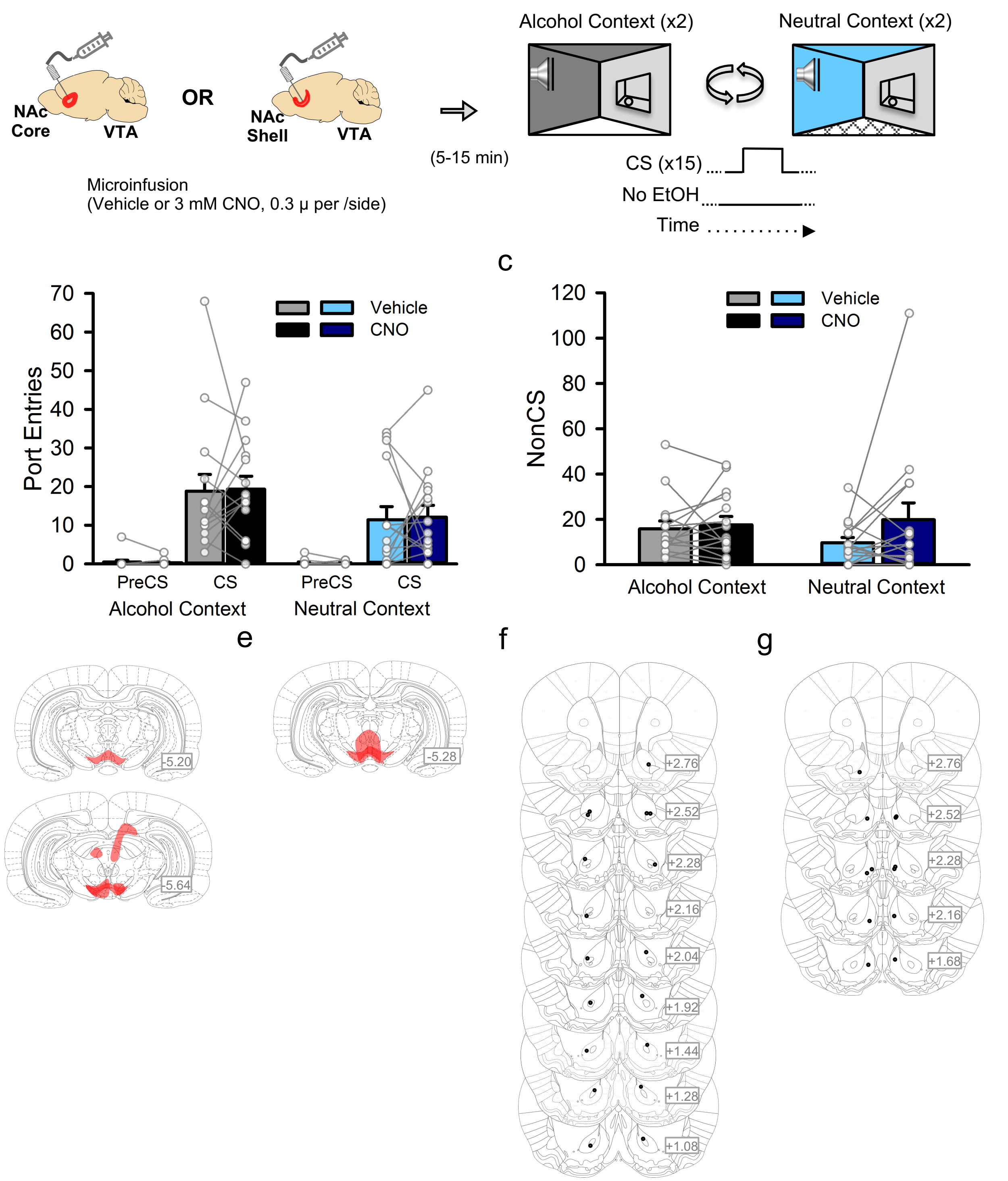

Supplementary Fig. 8 | Assessing off-target effects of CNO microinfusion in the nucleus accumbens core and shell. We examined whether clozapine-*n*-oxide (CNO) microinfusion in the nucleus accumbens (NAc) core or shell affected CS-triggered alcohol-seeking in an alcohol or neutral context in the absence of designer receptor expression. Wild-type rats (*n*=15) were implanted with cannulae targeting the NAc core (*n*=2 female; *n*=7 male) or shell (*n*=2 female;

n=4 male). Half of the rats in each group received a microinfusion of the AAV8-hSyn-DIO-mCherry construct in the VTA (NAc core, *n*=5; NAc shell, *n*=2) whereas the remainder did not. Rats then received 24 h access to a 15% ethanol solution in their home-cage every other day for a total of twelve sessions. Water and standard rat chow were continuously available. Rats received Pavlovian conditioning sessions every other day in a distinct alcohol context where a discrete, 10 s auditory conditioned stimulus (CS; 15 trials per session) was paired with 15% ethanol (0.2 ml per CS; 3.0 ml per session; variable inter trial interval = 260 s). On alternating days, rats were exposed to a different, neutral context where a distinct, 10 s neutral auditory stimulus (NS; 15 trials per session) was presented without alcohol. One rat received 2 sessions each of 15% ethanol that was adulterated with 2% sucrose, then 1% sucrose, during training and was maintained on a 15% ethanol solution adulterated with 0.5 sucrose thereafter to aid the acquisition of Pavlovian conditioning. **a** CS-triggered alcohol-seeking was tested 4 times per rat, twice in the alcohol context and twice in the neutral context by presenting the CS exactly as during training but without alcohol. Rats received a microinfusion of vehicle or CNO (3 mM, 0.3 μl) prior to each test. **b** At test, CS port-entries were elevated in the alcohol context relative to the neutral context [Context x Interval, $F_{(1, 13)}$=5.337, *p*=.038, η_p^2=.291]. CNO microinfusion had no impact on port-entries as the main effect of Treatment and all higher order interactions with treatment were nonsignificant (All *F*<1). Whether the rats were cannulated in the NAc core or NAc shell also had no impact on responding at test as the main effect of Region and all higher order interactions with region were nonsignificant (All *F*<1). **c** NonCS port-entries were similar across contexts at test as the main effect of Context and all higher order interactions with context were nonsignificant (All *F*<1.212, *p*>.291). CNO microinfusion had no impact on NonCS port-entries as the main effect of Treatment and all higher order interactions with treatment were nonsignificant (All *F*<2.085, *p*>.172). Whether rats were cannulated in NAc core or NAc shell also had no impact on NonCS port-entries at test as the main effect of Region and all higher order interactions with region were nonsignificant (All *F*<1). Histology results show mCherry expression in the midbrain for rats cannulated in the **d** NAc core and **e** NAc shell as well as corresponding injector tip placements in the **f** NAc core and **g** NAc shell. Averaged data are mean ± s.e.m. with data from individual rats overlaid on the bar graphs. Data were analyzed using RM ANOVA. All statistical tests were two-sided. Raw data are available as a supplementary source data file.

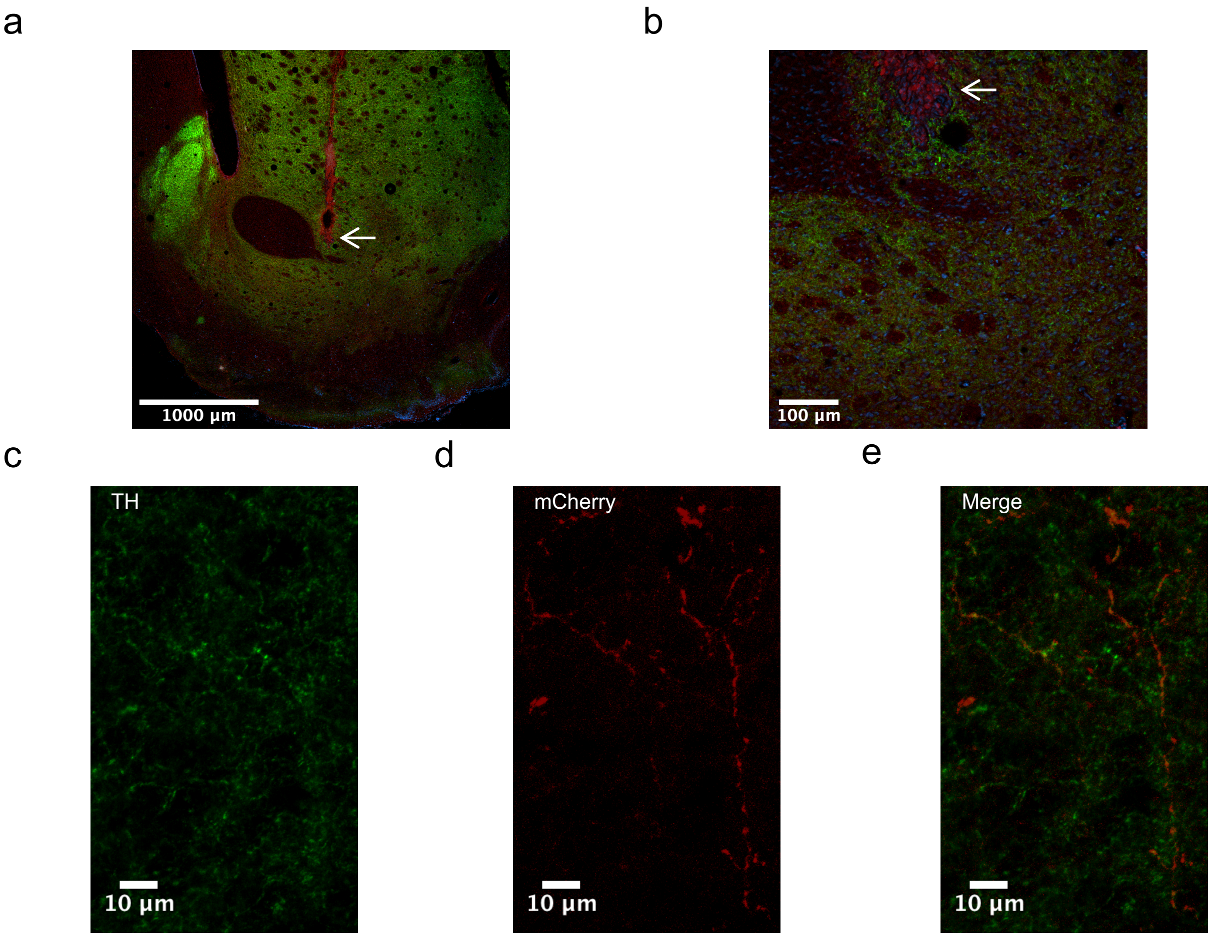

Supplementary Fig. 9│Immunofluorescence images from the nucleus accumbens (NAc) core. A representative coronal section of confocal images through the striatum showing fluorescence indicative of mCherry (red; reporter for hM4Di; amplified with Alexa 594), Tyrosine Hydroxylase (TH; green; Alexa 488), and the nuclear marker DAPI (blue). **a** A 4X image of the ventral striatum showing placement of an injector (arrow) that was used to microinfuse CNO in the NAc core. **b** A 20X image showing the lower-right quadrant of NAc nucleus accumbens core and injector placement (arrow). **c** A 60X image showing TH+ neural processes in the NAc core near the injector tip in the previous panels. **d** The same processes showing mCherry expression. **e** A merge of TH and mCherry expression showing signal overlap. All images in this figure are z-projections of maximum signal to highlight processes across the z-plane.

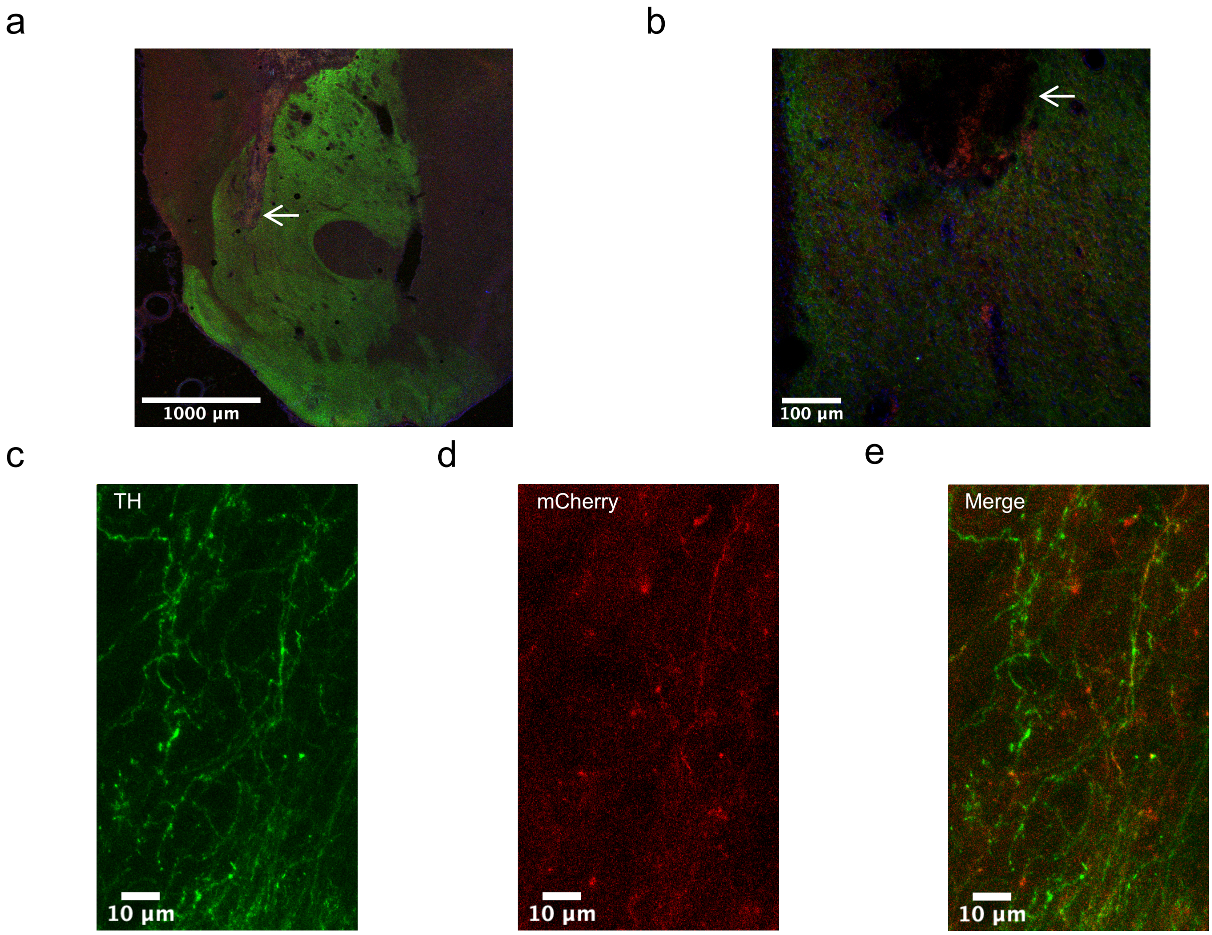

Supplementary Fig. 10│Immunofluorescence images from the nucleus accumbens (NAc) shell. A representative coronal section of confocal images through the striatum showing fluorescence indicative of mCherry (red; reporter for hM4Di; amplified with Alexa 594), Tyrosine Hydroxylase (TH; green; Alexa 488), and the nuclear marker DAPI (blue). **a** A 4X image of the ventral striatum showing placement of an injector (arrow) that was used to microinfuse CNO in the NAc shell. **b** A 20X image showing the injector tip placement (arrow) in the NAc shell. **c** A 60X image showing TH+ neural processes in the NAc shell near the injector tip in the previous panels. **d** The same processes showing mCherry expression. **e** A merge of TH and mCherry expression showing signal overlap. All images in this figure are z-projections of maximum signal to highlight processes across the z-plane.

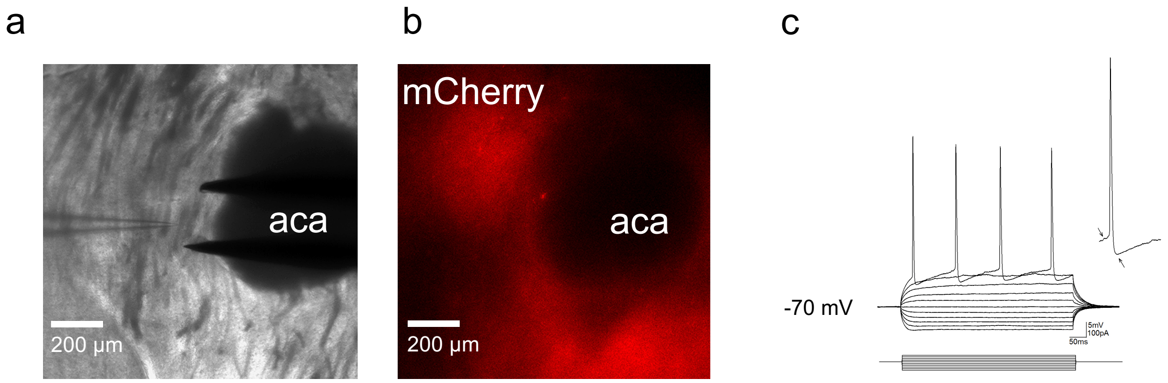

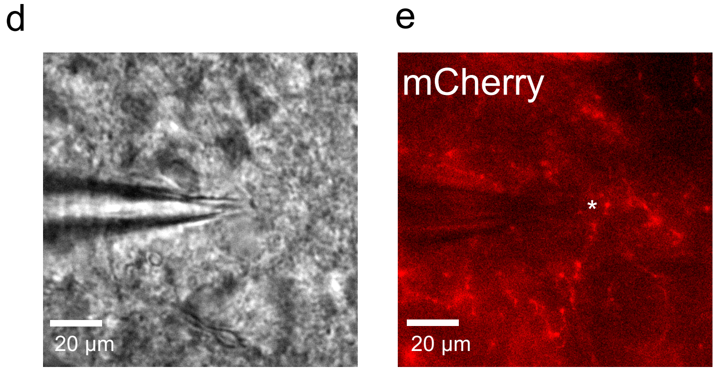

Supplementary Fig. 11 | Microscopy images obtained during electrophysiological recordings from nucleus accumbens (NAc) medium spiny neurons (MSN). a A representative brightfield image showing the position of stimulating and recording electrodes in the NAc core. **b** A representative fluorescence image of the same coronal slice showing strong mCherry expression in VTA fibers spread throughout the NAc core (aca: anterior portion of the anterior commissure). **c** Superimposed membrane potential responses in a representative MSN in response to hyperpolarizing and depolarizing current steps showing isolated action potentials. Arrows indicate the slow ramp-like depolarization prior to firing, and large fast after-hyperpolarization that are typical of MSN neurons. Resting membrane potential of MSNs was -66.1 ± .4 mV, capacitance was 23.4 ± 3.2 pF, and cellular input resistance was 188 ± 14.5 MΩ. A 5 min bath application of 1 µM CNO to inhibit hM4Di-expressing terminals of VTA neurons within the NAc core did not produce significant changes in action potential number (2.4 ± .5 at baseline vs. 1.7 ± .5 in CNO, t=1.18, p=0.28), width (9.7 ± .7 ms at baseline vs. 9.5 ± .7 ms in CNO, t=1.76, p=0.14), or height (93.9 ± 3.4 mV at baseline vs. 94.6 ± 3.9 mV in CNO, t=0.23, p=0.83) during injection of positive current steps. However, CNO acting on inhibitory designer receptors on dopamine terminals significantly lowered action potential threshold from -43.2 ± 1.5 mV at baseline to -49.7 ± 2.4 in CNO (p=.002), which is consistent with the observation that dopamine agonists can shift threshold to more depolarized voltages[108]. CNO also resulted in a small decrease in the fast afterhyperpolarization (5.7 ± 0.7 mV at baseline to 4.6 ± 0.7 in CNO, p=.017). **d** Image of an MSN that was recorded from. **e** Image of the same MSN in **d** relative to mCherry-expressing dopaminergic VTA fibers. Data were analyzed using RM ANOVA and Bonferroni-corrected t-tests. All tests were two-sided.

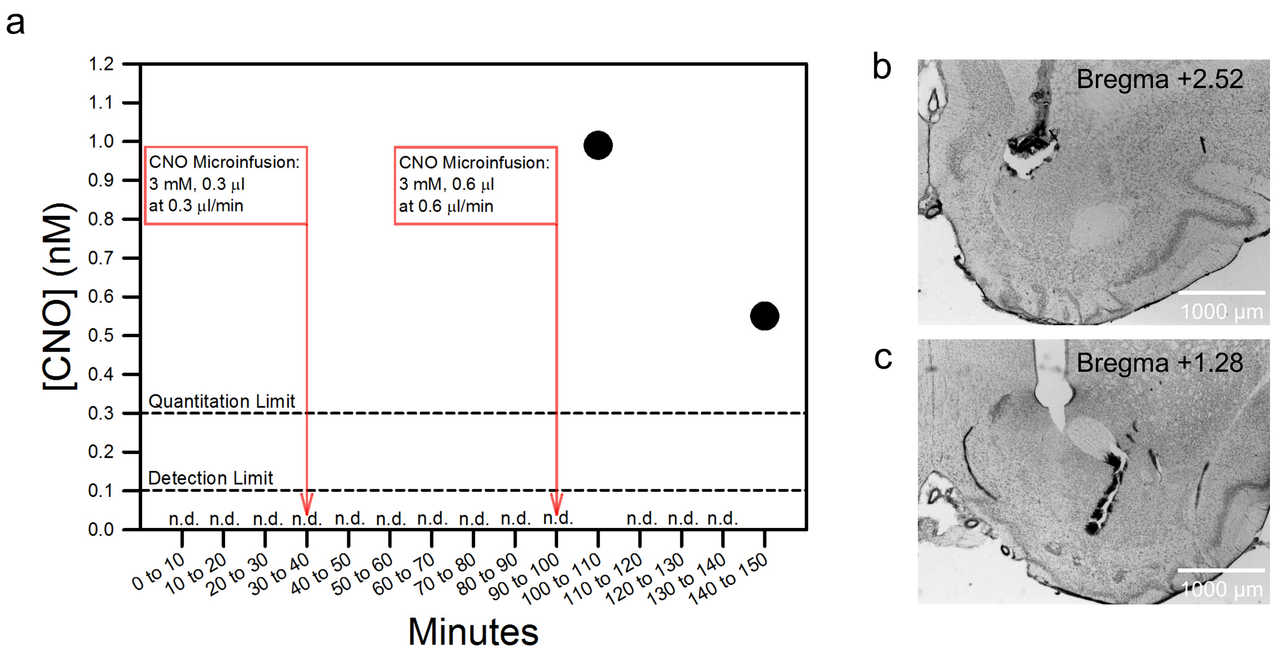

Supplementary Fig. 12│Assessing the diffusion of CNO from the NAc core to NAc shell following microinfusion. a Quantification of clozapine-*n*-oxide (CNO) in dialysate samples using high performance liquid chromatography mass spectrometry (HPLC-MS). **b** A nissl stained 2.5X light microscopy image of the NAc showing damage indicating the location of the microdialysis probe and **c** the location of CNO microinfusions. Microdialysis and CNO detection methods: A rat was anesthetized (isoflurane; 5% induction, 2-3% maintenance) and secured in a stereotaxic frame. A 21G cannula was implanted targeting the NAc shell (in mm at a 10° angle, AP: 1.68, ML: ±2.23, DV: -4.85 mm) and secured in place with dental cement. A probe made with semi-permeable membrane (1.5 mm active membrane exposed; 280 µm O.D.; 6% recovery of CNO) was lowered down the cannula (protruding 3 mm) and left in place for 150 min while artificial cerebrospinal fluid (aCSF) was perfused through the membrane (2 µl/min) and collected (20 µl per 10 min). At 30 minutes after the probe was inserted in the brain, a CNO microinfusion (3 mM, .3 µl, .3 µl per min) was made in the NAc core using modified coordinates that targeted the same area where CNO was microinfused in our previous experiments (in mm at 16° angle, AP: 1.2, ML: ±4, DV -7.28). Before the infusion began the injector was lowered to -7.48 mm and retracted up to -7.28 mm for the microinfusion to mimic the clearing of tissue that normally occurs during saline habituation microinfusions. At 90 min into the collection of dialysate a second CNO microinfusion in the NAc core was conducted at double the volume and rate (3 mM, .6 µl, .6 µl per min). Dialysate samples were collected for 60 min following the first and the second microinfusions. After the last dialysate sample was collected the rat was sacrificed. Determination of CNO was performed with

an LC-MS instrument. Chromatographic separation was carried out using an HPLC system (Agilent 1200 HPLC) equipped with a Biphenyl column (Kinetex® 2.6 µm Biphenyl 100 Å, LC Column 50 x 2.1 mm, Phenomenex, Tarrance, CA). Solvent used, A : Formic Acid 0.01% with 2 mM Ammonium Acetate, B : Methanol. The gradient program began at 40% A and changed as follows: 6 min 70%, 7 min 95% for 1 min and back to 40% at 10 min with constant flow rate of 0.25 ml per min. From the 20 µl of sample collected, 10 µl were transferred to a conical vial insert and 2 µl of internal standard were added and mixed. 10 µl of this solution were injected on the column. The internal standard selected was Clozapine-D4, which eluted 1 min before CNO, and this signal compensated for variability of MS measurements. The mass spectrometer was a QTOF ULTIMA (Waters Canada, Mississauga, Ontario.) equipped with an electrospray interface (ESI) set in the positive (ESI+) mode. Raw data are available as a supplementary source data file.
