# Supplementary material for: Dissociable mesolimbic dopamine circuits control responding triggered by alcohol-predictive discrete cues and contexts
Source: Nat Commun. 2020 Jul 28;11:3764. doi: 10.1038/s41467-020-17543-4 (PMC7534644; doi:10.1038/s41467-020-17543-4)
Supplement: Supplementary file 2 — Description of Additional Supplementary Files [file 41467_2020_17543_MOESM2_ESM.pdf]

Description of Additional Supplementary Files

File Name: Supplementary Movie 1
Description: shows the same rat responding by making CS port entries in the alcohol context on the top panel and in the neutral context on the bottom panel.
