# Supplementary material for: Dissociable mesolimbic dopamine circuits control responding triggered by alcohol-predictive discrete cues and contexts
Source: Nat Commun. 2020 Jul 28;11:3764. doi: 10.1038/s41467-020-17543-4 (PMC7534644; doi:10.1038/s41467-020-17543-4)
Supplement: Supplementary file 4 — Reporting Summary [file 41467_2020_17543_MOESM4_ESM.pdf]

nature research

Corresponding author(s): Dr. Nadia Chaudhri

Last updated by author(s): June 8th 2020

Reporting Summary

Nature Research wishes to improve the reproducibility of the work that we publish. This form provides structure for consistency and transparency in reporting. For further information on Nature Research policies, see our Editorial Policies and the Editorial Policy Checklist.

Statistics

For all statistical analyses, confirm that the following items are present in the figure legend, table legend, main text, or Methods section.

n/a	Confirmed	
☐	☒	The exact sample size (*n*) for each experimental group/condition, given as a discrete number and unit of measurement
☐	☒	A statement on whether measurements were taken from distinct samples or whether the same sample was measured repeatedly
☐	☒	The statistical test(s) used AND whether they are one- or two-sided<br>*Only common tests should be described solely by name; describe more complex techniques in the Methods section.*
☒	☐	A description of all covariates tested
☐	☒	A description of any assumptions or corrections, such as tests of normality and adjustment for multiple comparisons
☐	☒	A full description of the statistical parameters including central tendency (e.g. means) or other basic estimates (e.g. regression coefficient) AND variation (e.g. standard deviation) or associated estimates of uncertainty (e.g. confidence intervals)
☐	☒	For null hypothesis testing, the test statistic (e.g. *F*, *t*, *r*) with confidence intervals, effect sizes, degrees of freedom and *P* value noted<br>*Give P values as exact values whenever suitable.*
☒	☐	For Bayesian analysis, information on the choice of priors and Markov chain Monte Carlo settings
☒	☐	For hierarchical and complex designs, identification of the appropriate level for tests and full reporting of outcomes
☐	☒	Estimates of effect sizes (e.g. Cohen's *d*, Pearson's *r*), indicating how they were calculated

Our web collection on statistics for biologists contains articles on many of the points above.

Software and code

Policy information about availability of computer code

Data collection	Data was collected using MED-PC v4.0 (Med Associates), NIS Elements (Nikon), CellSens v1.8 (Olympus), Fiji v2.0, Clampfit v8.2 and pClamp v10.3 (both Molecular Devices).
Data analysis	Statistical analyses were conducted with SigmaPlot v12 or SPSS v20 and graphs were made with SigmaPlot v12.

For manuscripts utilizing custom algorithms or software that are central to the research but not yet described in published literature, software must be made available to editors and reviewers. We strongly encourage code deposition in a community repository (e.g. GitHub). See the Nature Research guidelines for submitting code & software for further information.

Data

Policy information about availability of data

All manuscripts must include a data availability statement. This statement should provide the following information, where applicable:
- Accession codes, unique identifiers, or web links for publicly available datasets
- A list of figures that have associated raw data
- A description of any restrictions on data availability

Raw data is available as a supplementary source data file. Protocols will be made available by the corresponding author upon request.

Field-specific reporting

Life sciences study design

All studies must disclose on these points even when the disclosure is negative.

Sample size	Sample size was not statistically predetermined, but based on common practice, previous studies (Millan et al., 2015, Neuropsychopharmacology; Sciascia et al., 2015, Neuropsychopharmacology), and the yield of our transgenic breeding facility.
Data exclusions	Out of a total of 139 rats, 12 failed to acquire Pavlovian conditioning, 9 had missed cannula placements, 1 had a brain lesion from infection in the target region, 2 experienced a programming error, and 1 had the house-light burn out at test. Data from these rats were excluded.
Replication	The main behavioural finding that CS port-entries were elevated in the alcohol context relative to the neutral context was replicated across 4 separate experiments within the manuscript (Exp. 1a, 5, 6 and 7). Any experiments conducted in serial replicates were replicated successfully and are noted in the manuscript.
Randomization	In within-subjects studies animals were assigned, based on previous alcohol consumption, to receive certain configurations of stimuli to achieve a counterbalanced design. In between-subjects experiments animals were assigned to groups to balance previous levels of alcohol consumption.
Blinding	Experimenters were blind to group for between-subjects experiments. However, most experiments were within-subjects designs in which all subjects received all conditions, and conditions were somewhat identifiable (e.g. treatment solutions or contexts were identifiable) which precluded a complete blinding of experimenters. Importantly, the main dependent measures (e.g. port entries) were recorded by automated software and could not be influenced by the experimenter.

Reporting for specific materials, systems and methods

We require information from authors about some types of materials, experimental systems and methods used in many studies. Here, indicate whether each material, system or method listed is relevant to your study. If you are not sure if a list item applies to your research, read the appropriate section before selecting a response.

Materials & experimental systems

n/a	Involved in the study	
☐	☒	Antibodies
☒	☐	Eukaryotic cell lines
☒	☐	Palaeontology and archaeology
☐	☒	Animals and other organisms
☒	☐	Human research participants
☒	☐	Clinical data
☒	☐	Dual use research of concern

Methods

n/a	Involved in the study	
☒	☐	ChIP-seq
☒	☐	Flow cytometry
☒	☐	MRI-based neuroimaging

Antibodies

Antibodies used	Rabbit anti-TH antibody EMD Milipore #AB152<br>Mouse anti-mCherry antibody Abcam #ab125096<br>Donkey anti-mouse IgG (H+L) Alexa 594 Jackson ImmunoResearch labs #715-585-150<br>Donkey anti-rabbit IgG (H+L) Alexa 488 Jackson ImmunoResearch labs #711-545-152
Validation	The specificity of the primary anti-TH and anti-mCherry antibodies was validated by the manufacturer and in publications listed on the manufacturer website (e.g., Edri et al., Nat Commun, 2015; Scrivo et al., Nat Commun, 2019). Additionally we only observed positive TH and mCherry signal in brain areas that are known to contain TH positive cell bodies and projections. In tissue that did not express mCherry, we failed to detect signal from the secondary anti-mCherry antibody.

Animals and other organisms

Policy information about studies involving animals; ARRIVE guidelines recommended for reporting animal research

Laboratory animals	Twenty-five wild-type male Long-Evans (220-275 g on arrival, INVIGO), 20 wild-type outbred (4 female, 15 male) Long-Evans (bred in-house) and 94 outbred male, Long-Evans, TH::Cre+/- rats (bred in-house) were used in the current study. Founder TH::Cre rats were generously provided by Dr. Karl Deisseroth (Witten et al., Neuron, 2011). All rats were 2-3 months of age at the beginning of experiments.
Wild animals	Wild animals were not used in this study.
Field-collected samples	Field-collected samples were not used in this study.

Ethics oversight	Procedures were approved by the Animal Research Ethics Committee at Concordia University and complied with guidelines from the Canadian Council on Animal Care.

Note that full information on the approval of the study protocol must also be provided in the manuscript.
